# Integrated cell-free DNA and cytokine analysis uncovers distinct tissue injury and immune response patterns in solid organ transplant recipients with COVID-19

**DOI:** 10.21203/rs.3.rs-1262270/v1

**Published:** 2022-01-20

**Authors:** Temesgen E. Andargie, Weiqiang Zhou, Andrew H. Karaba, Taibo Li, Fayaz Seifuddin, Alex G. Rittenhouse, Hyesik Kong, Komudi Singh, Robert Woodward, Aldo Iacono, Robin K Avery, Mehdi Pirooznia, Moon Kyoo Jang, Hongkai Ji, Andrea L. Cox, Sean Agbor-Enoh

**Affiliations:** 1 Genomic Research Alliance for Transplantation (GRAfT) and Laboratory of Applied Precision Omics, National Heart, Lung, and Blood Institute (NHLBI), NIH, Bethesda, MD.; 2 Department of Biology, Howard University, Washington DC.; 3 Department of Biostatistics, Bloomberg School of Public Health, Johns Hopkins University, Baltimore, MD.; 4 Department of Medicine, Johns Hopkins University, School of Medicine, Baltimore, MD.; 5 Bioinformatics and Computation Core, NHLBI, MD.; 6 CareDx, Brisbane, CA.; 7 Department of Medicine, University of Maryland, College Park, MD.

## Abstract

COVID-19 pathogenesis is associated with an exuberant inflammatory response. However, the tissue injury pattern and immune response in solid-organ transplant recipients (SOTRs) taking immunosuppressive therapy have not been well characterized. Here, we perform both cfDNA and cytokine profiling on plasma samples to map tissue damage, including allograft injury and delineate underlying immunopathology. We identified injuries from multiple-tissue types, including hematopoietic cells, vascular endothelium, hepatocyte, adipocyte, pancreas, kidney, heart, and lung in SOTRs with COVID-19 that correlates with disease severity. SOTRs with COVID-19 have higher plasma levels of cytokines such as IFN-λ1, IFN-γ, IL-15, IL-18 IL-1RA, IL-6, MCP-2, and TNF-α as compared to healthy controls, and the levels of GM-CSF, IL-15, IL-6, IL-8, and IL-10 were associated with disease severity in SOTRs. Strikingly, IFN-λ and IP-10 were markedly increased in SOTRs compared to immunocompetent patients with COVID-19. Correlation analyses showed a strong association between monocyte-derived cfDNA and inflammatory cytokines/chemokines in SOTRs with COVID-19. Moreover, compared to other respiratory viral infections, COVID-19 induced pronounced injury in hematopoietic, vascular endothelial and endocrine tissues. Allograft injury, measured as donor-derived cfDNA was elevated in SOTRs with COVID-19, including allografts distant from the primary site of infection. Allograft injury correlated with inflammatory cytokines and cfDNA from immune cells. Furthermore, longitudinal analysis identified a gradual decrease of cfDNA and inflammatory cytokine levels in patients with a favorable outcome. Our findings highlight distinct tissue injury and cytokine features in SOTRs with COVID-19 that correlate with disease severity.

## 1. Introduction

Advances in solid organ transplantation (SOT) have resulted in improved survival in patients with end-stage organ failure, and there are nearly 1.5 million people living with organ transplants worldwide^1^. However, viral infections after SOT are a major cause of severe disease and reduced allograft and patient survival^2–4^, due to immunosuppression and a high prevalence of comorbidities. The current COVID-19 pandemic continues to threaten the world with over 275 million cases and 5.3 million deaths as of December 21, 2021 (https://coronavirus.jhu.edu/). Similar concerns exist in SOT recipients (SOTRs) infected with SARS-CoV-2. The clinical spectrum of COVID-19 in transplant patients has been variable in published reports, ranging from mild disease to severe multiple-organ failure and death^3,5–7^. However, the tissue source of injury that contributes to these variable clinical trajectories remains poorly defined. The immunogenicity of COVID-19 vaccines is suboptimal in SOTR^8–10^ and new SARS-CoV-2 variants continue to emerge^11^, which is worsening the situation. It has also been shown in some studies that immunosuppressive drug mycophenolate mofetil (MMF) is linked to severe COVID-19 outcome^12^and to lower antibody responses after mRNA vaccines in SOTRs^13^. It is therefore critical to develop approaches that map the tissue source of injury (including allograft), the pathogenesis, and predict long-term outcomes. Such approaches may guide optimal timing to initiate aggressive treatment towards reducing severe disease in SOTRs.

Circulating cell-free DNA (cfDNA), released into the bloodstream from cell injury or death, is a promising noninvasive biomarker for the diagnosis and monitoring of different disease ststes^14–18^. Healthy individuals have small amounts of cfDNA, predominantly arising from normal cell turnover of hematopoietic cell lineages^19^. However, in individuals with pathological conditions, the concentration of cfDNA is elevated, with a significant amount of cfDNA emanating from the affected tissues^19^. In SOT patients, allograft injury occurs resulting in elevated donor-derived cfDNA (ddcfDNA) in the recipient’s circulation, released from allografts. Quantifying ddcfDNA by targeting single-nucleotide polymorphisms (SNPs) has become a promising clinical tool for real-time monitoring of allograft health^20,21^. Indeed, we have demonstrated ddcfDNA as a sensitive biomarker to detect allograft rejection and predict long-term outcomes^22,23^. In addition, cfDNA also carries cell-type-specific epigenetic methylation signatures and chromatine footprints. Recent studies used genome-wide DNA methylation patterns to deconvolute cfDNA tissues of origin and infer cell death in specific tissue or organ types^19,24–26^. In the general population with COVID-19 patients, we and others have reported markedly elevated cfDNA levels from divergent tissue types that correlated with disease progression and outcome^27,28^. However, the effect of SARS-CoV-2 infections on the allograft and recipient tissue cell and tissue types of SOTRs with COVID-19 is not completely delineated.

The pathogenesis of COVID-19 is multifactorial, involving direct viral infection, a hyperinflammatory response, immunothrombosis, and coagulopathy^29^. SARS-CoV-2 uses angiotensin-converting enzyme 2 for cell entry, which is expressed in multiple tissue types, including lung, heart, kidney and blood vessels^30^. Notably, most of the severe COVID-19 complications that result in multiorgan failure and death are primarily attributed to virus-independent immunopathologic mechanisms^30^. Indeed, high serum levels of inflammatory cytokines such as interleukin-6 (IL-6), IL-8, and tumor necrosis factor-α (TNF-α) are observed in COVID-19 patients and predicted disease severity and poor outcome^31,32^. Additionally, cfDNA is also considered as a trigger of deleterious proinflammatory and prothrombotic pathways in addition to being a biomarker^33–36^. In SOTRs, the immunosuppressive drugs taken to prevent allograft rejection may be beneficial in suppressing this hyperinflammatory state and protecting severe forms of COVID-19, but this could also diminish host defense against the virus. However, studies comparing the relationship between tissue injury patterns and immune response in SOTRs with COVID-19 and immunocompetent hosts are scarce.

In this study of SOTRs with COVID-19, we provide evidence of significant tissue injury that correlated with disease severity. Using whole-genome bisulfite sequencing and a library of tissue-specific DNA methylation markers, we show that COVID-19 induces injury for multiple tissue types, including transplanted organs that correlated with disease severity. We also show a comparable tissue injury profile between SOTRs with COVID-19 and Non-SOT COVID-19 patients matched for disease severity. In addition, we characterized the immune response using a multiplex cytokine assay and correlated it with cfDNA profiles. SOTRs with COVID-19 have distinct cytokine signatures that are strongly correlated with myeloid-derived cfDNA profiles. We further reveal COVID-19 specific tissue injury characteristics in lung transplant recipients (LTRs) as compared to other respiratory viral infections (other RVIs). Finally, we demonstrated that longitudinal cfDNA and proinflammatory cytokine levels remained elevated in a patient who eventually died while gradually decreasing in recovered patients. Taken together, these data identify distinct mechanisms of pathology in SOTRs and highlight integrative analysis of cfDNA and cytokine signatures as a comprehensive molecular marker of tissue injury and inflammation that may help predict disease severity in SOTRs with COVID-19.

## 2. Results

### Demographics and clinical characteristics of SOT recipients with COVID-19

**Figure 1.**
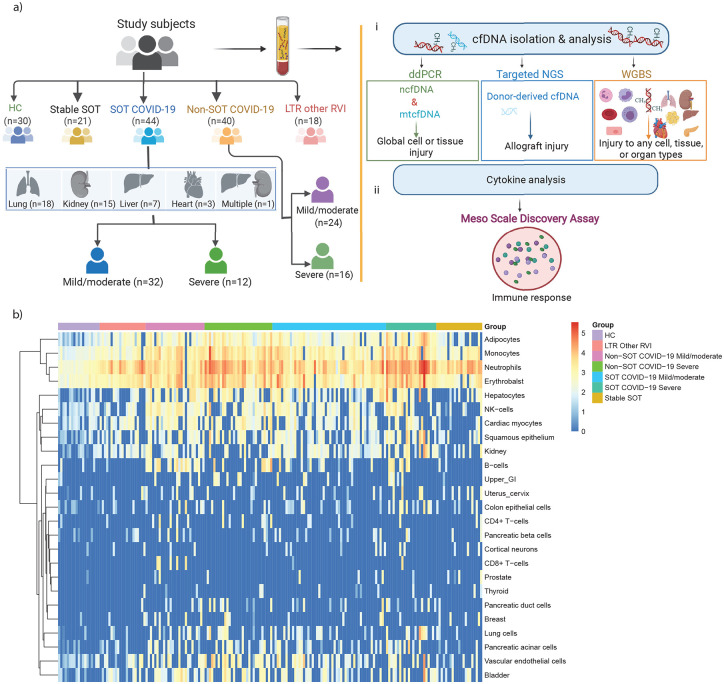
Study design and experimental workflow. **a**. Schematic diagram of study subjects (including 30 Healthy controls [HC], 21 Stable solid organ transplant [SOT] controls, 44 SOT COVID-19 [32 Mild/moderate and 12 severe] patients, 40 Non-SOT COVID-19 [24 Mild/moderate and 16 severe] patients, and 18 Lung transplant recipients with other respiratory viral infections [Other RVIs]). **b.** Schematic representation of plasma cfDNA and cytokine quantification. **c.** Heatmap representation of tissue-specific cfDNA profile across the groups.

A total of 44 consecutive SOTRs requiring hospitalization with confirmed COVID-19 were included: 18 (40.9%) lung, 16 (36.4%) kidney, 5 (11.6%) liver, 3 (6.8%) heart, and 1 (2.3%) combined kidney-liver recipients (Fig.1a). The median age was 54.5 years (interquartile range [IQR]: 43.5 – 66.5), the median time from transplantation was four years (IQR: 1–8.5), and 28 (61.4%) patients were male. The median body mass index (BMI) was 28.3 kg/m^2^ (IQR: 24.15 – 34.28). The most common comorbidities were hypertension (n=27, 61.4%) and diabetes (n=21, 47.7%). The median length of stay in the hospital was eight days (IQR: 6–20), and all the SOTRs were on an immunosuppressive regimen. Thirty patients (68.2%) were on a regimen containing mycophenolate prior to COVID-19 diagnosis, and it was discontinued in the majority of the patients (n=27; 90%) upon admission. All patients received tacrolimus. Among the 44 SOTRs, 12 (31.5%) patients developed severe COVID-19 with ICU admission during their disease course and 32 patients had mild/moderate disease and eventually recovered. Compared to patients with mild/moderate disease, those who developed severe disease were older (median age 62.5 vs. 50 years, p=0.03), and on admission had higher WBC count (7.5 vs. 4.7 k/uL, p=0.015), absolute neutrophil count (ANC) (5.9 vs. 3.1 k/uL, p=0.002), and D-dimer (2.57 vs. 0.47 mg/L, p=0.001) upon admission. There also had longer hospitalization stay (26.5 vs. 7 days, p<0.001). More than half of COVID-19 patients were treated with remdesivir (n=23, 52.3%) and 45% of the patients received convalescent plasma. Three of the 12 SOTRs with severe disease died due to complications of COVID-19. Baseline demographic and clinical characteristics of the SOT patients are shown in table 1. The study also included 40 Non-SOT COVID-19 patients to investigate whether the tissue injury pattern is different from COVID-19 SOTRs. Non-SOT COVID-19 group was similar to SOT COVID-19 regarding demographics, disease severity scores, need for ICU care and survival (Suppl.Table 1). In addition, we collected plasma samples from 18 (21 episodes) lung transplant recipients (LTRs) with other respiratory viral infections (other RVIs: influenza and respiratory syncytial virus), 21 stable transplant patients with no history of infection and rejection and from 19 healthy controls (HCs) for comparison purposes.

#### cfDNA maps COVID-19 associated tissue injury in SOTRs.

Given the ongoing nature of the COVID-19 pandemics and the high risk of severe complications in immunocompromised hosts, a comprehensive tissue-damage mapping in transplant patients infected with COVID-19 is a pressing need to illustrate the extent of tissue injury that correlates with disease severity and outcomes. To do that, we obtained admission plasma samples from 44 SOT COVID-19 patients, as well as plasma from stable SOTRs (n=21) and healthy controls (HCs) (n=19). We first isolated and quantified total plasma cell-free mitochondrial (mtcfDNA) and nuclear DNA (ncfDNA), measures of global cellular death or tissue injury, via digital droplet PCR using mitochondrial and nuclear target primer sets, respectively. cfDNA showed a nucleosomal distribution with a prominent peak length around ~167 bp (Supp.Fig.1), indicating good quality of cfDNA. We next performed whole-genome bisulfite sequencing and used DNA methylation-based deconvolution algorithm to identify and determine the relative contribution of each tissue type to the plasma cfDNA^19^. We then computed the absolute copies of tissue-specific cfDNA per mL plasma (cp/mL) by multiplying the relative estimated proportions of tissue-specific cfDNA with ncfDNA (cp/mL) (see Methods) and generated heatmap on log-transformed cfDNA data. The heatmap revealed divergent tissue injury patterns in both SOT and Non-SOT COVID-19 patients (Fig.1b).

**Figure 2.**
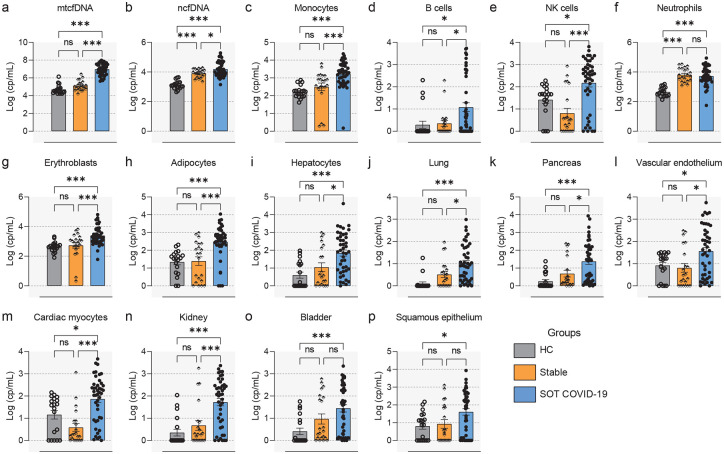
Elevation of circulating cfDNA levels in solid organ transplant recipients (SOTRs) with COVID-19. Comparison of absolute plasma cell-free mitochondrial (mtcfDNA) (**a**) and nuclear DNA (ncfDNA) (**b**) and tissue-specific cfDNA derived from monocytes (**c**), B cells (**d**), NK cells (**e**), neutrophils (**f**), erythroblasts (**g**), vascular endothelium (**h**), adipocytes (**i**), hepatocytes (**j**), lung (**k**), pancreas (**l**), cardiac myocytes (**m**), kidney (**n**), bladder (**o**), and squamous epithelium (**p**) from Healthy controls (HCs) (n=19), stable SOT controls (n=21), and SOTRs with COVID-19 patients (n=44). Plasma ncfDNA and mtcfDNA concentrations were measured by digital droplet PCR. cfDNA Whole-genome bisulfite sequencing was performed to measure tissue-specific cfDNA profiles, leveraging tissue-specific DNA methylomes and deconvolution algorithms. Median [interquartile range (IQR)] of cfDNA copies per mL (cp/mL) are reported. Statistical significance was determined by the Mann–Whitney test. Adjusted p values are reported (for multiple comparison and demographic factors (age, sex, and BMI). A p-value ≤0.05 and FDR≤0.25 was considered statistically significant;*: FDR ≤ 0.25 and p-value ≤ 0.05, **: FDR ≤ 0.1 and p-value ≤ 0.05, ***: FDR ≤ 0.05 and p-value ≤ 0.05, NS: FDR > 0.25 or p-value > 0.05.

**Figure 3.**
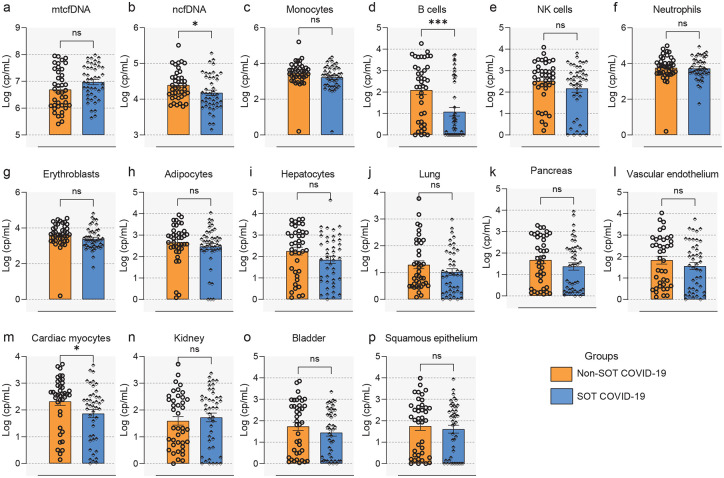
Comparable cfDNA profiles between SOT and Non-SOT COVID-19 patients. Absolute plasma mtcfDNA (**a**) and ncfDNA (**b**) levels in Non-SOT (n=40) and SOT COVID-19 patients. c–p. Quantification of tissue-specific cfDNA levels: monocytes (**c**), B cells (**d**), NK cells (**e**), neutrophils (**f**), erythroblasts (**g**), vascular endothelium (**h**), adipocytes (**i**), hepatocytes (**j**), lung (**k**), pancreas (**l**), cardiac myocytes (**m**), kidney (**n**), bladder (**o**), and squamous epithelium (**p**) from Non-SOT and SOT COVID-19 patients. Statistical significance was determined by the Mann–Whitney test. Adjusted p values are reported (for multiple comparison and demographic factors (age, sex, and BMI). A p-value ≤0.05 and FDR≤0.25 was considered significant;*: FDR ≤ 0.25 and p-value ≤ 0.05, **: FDR ≤ 0.1 and p-value ≤ 0.05, ***: FDR ≤ 0.05 and p-value ≤ 0.05, NS: FDR > 0.25 or p-value > 0.05.

The results of our analyses revealed markedly elevated mtcfDNA (median [IQR] = 10,361,850 [3,076,139 – 32,192,441] cp/mL) levels in SOTRs with COVID-19 compared to stable transplant (median [IQR] = 105,730 [51,158 – 30,4126] cp/mL) or healthy controls (median [IQR] = 30,334 [22,995 – 66,624] cp/mL) (p<0.05 and FDR<0.25; Fig.2a). Similarly, the absolute copy number of ncfDNA was significantly increased in SOT patients with COVID-19 (median [IQR] = 12,943 [6,502 – 41,546] cp/mL) compared to stable transplant (median [IQR] = 7,485 [4,646 – 23,701] cp/mL) or healthy controls (median [IQR] = 1,218 [891 – 1,870] cp/mL) (p<0.05 and FDR<0.25; Fig.2b). Our deconvolution analysis revealed the majority of circulating cfDNA are derived from hematopoietic origin cells. Specifically, cfDNA derived from monocytes, NK cells, B-cells, neutrophils and erythroblasts were significantly increased in SOTRs with COVID-19 compared to stable transplant and healthy controls (p<0.05 and FDR <0.25; Fig.2c–g). Likewise, we detect significant injuries from non-hematopoietic tissues (p<0.01 and FDR<0.25; Fig.2h–p), including adipocytes, hepatocytes, lung, pancreas, vascular endothelium, cardiac myocytes, and kidney in the bloodstream of SOT COVID-19 patients than in stable transplant and healthy controls. The levels of neutrophil, bladder and squamous epithelium derived cfDNA were significantly increased in SOT COVID-19 patinets relative to healthy controls, but not relative to stable transplant patients. Notably, total cfDNA and neutrophil-derived cfDNA were significantly higher in stable transplant patients compared to healthy controls (p<0.05 and FDR<0.25; Fig.2b and f), suggesting ongoing tissue injury imposed by the transplant state. This suggests that COVID-19 caused systemic injury to multiple cell and tissue types in SOTR with COVID-19. Additionally, our data showed no significant difference in cfDNA levels among SOT types (Suppl.Fig.2a–p). Further, tissue-specific cfDNA measures correlate with established markers of tissue or end-organ injury (Suppl.Fig.3) (see supplementary text for details).

#### Comparable cfDNA profiles between SOT and Non-SOT COVID-19 patients.

To examine the effect of COVID-19 on immunosuppressed transplant recipients, we compared cfDNA patterns in these patients to Non-SOT patients matched for COVID-19 disease severity median [IQR]: 4 (3–5) vs 4 (3–7), p=0.087). During the COVID-19 illness, SOTs were maintained on tacrolimus and mycofenalate was discontinued. We found that SOT patients with COVID-19 had significantly lower absolute copy number of ncfDNA when compared to those Non-SOT matched COVID-19 patients (median [IQR]: 12,943 [6,502 – 41,546] cp/mL vs 20,979 [12,312 – 42,054] cp/mL; p=0.035 and FDR=0.25), but not mtcfDNA copy number (Fig.3a–b). We then analyzed the level of tissue-specific cfDNA to measure tissue injury patterns (Fig.3c–p). The levels of B cell and cardiac myocyte-derived cfDNA were significantly lower in the SOTRs with COVID-19 as compared with the matched Non-SOT COVID-19 patients (p<0.05 and FDR<0.25; Fig.3d and m). We observed similar injury patterns with the other majority of cell and tissue types, including monocytes, NK cells, neutrophil, erythroblast, vascular endothelium, adipocytes, hepatocytes, lung and kidney derived cfDNA, when comparing between SOT patients with COVID-19 and Non-SOT COVID-19 comparator groups, although the latter tended to have higher levels of cfDNA (p>0.05 and FDR>0.25). This suggests a comparable tissue injury pattern between SOT and Non-SOT COVID-19 patients.

#### Distinct cytokine response in SOTRs with COVID-19.

**Figure 4.**
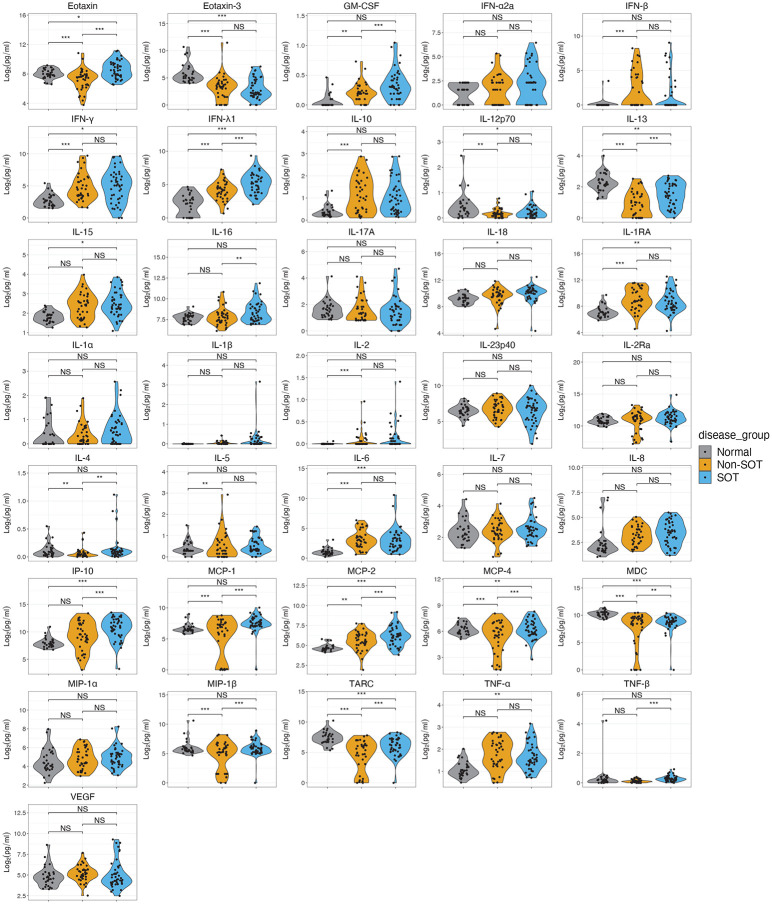
SOTRs with COVID-19 displayed dysregulated cytokine responses. Comparison of plasma cytokine/chemokine levels on healthy controls (n=30), Non-SOT COVID-19 patients (n=38) and SOTRs with COVID-19 (n=44). Cytokine values are reported as picograms per milliliter (pg/mL). Statistical significance was determined by the Mann–Whitney test. Adjusted p values are reported (for multiple comparison and demographic factors (age, sex & BMI). A p-value ≤0.05 and FDR≤0.25 was considered significant;*: FDR ≤ 0.25 and p-value ≤ 0.05, **: FDR ≤ 0.1 and p-value ≤ 0.05, ***: FDR ≤ 0.05 and p-value ≤ 0.05, NS: FDR > 0.25 or p-value > 0.05.

An excessive and dysregulated cytokine response is associated with severe COVID-19 disease^31,37^. To determine whether the immunosuppressed SOTRs with COVID-19 display dysregulated circulatory cytokines signatures, admission plasma levels of 36 cytokines and chemokines were measured (see Methods) in SOTRs with COVID-19 (n=44) and compared with matched Non-SOT COVID-19 patients (n=38) and healthy controls (n=30). We found altered cytokine levels in both SOT and Non-SOT patients with COVID-19 compared to healthy controls (p<0.05 and FDR<0.25; Fig.4). Specifically, high plasma levels of IFN-λ1, IFN-γ, IL-1RA, IL-6, MCP-2, and lower levels of Eotaxin-3, IL-12p70, IL-13, MCP-4, MDC, TARC, were observed both in SOT and Non-SOT COVID-19 patients as compared to HCs. The levels of IP-10, IL-15, IL-18, and TNF-α were exclusively increased in SOTRs with COVID-19. Conversely, significantly higher levels of GM-CSF, IFN-β, IL-10, and IL-2 and low levels of Eotaxin, IL-4, IL-5, MCP-1, and MIP-1β, were distinctive to Non-SOT COVID-19 patients relative to HCs, but not in SOTRs. The levels of IFN-β (p=0.059), GMCSF (p=0.099) and IL-10 (p=0.077) were higher in SOTRs, whereas in Non-SOT patients IL-1β (p=0.053), IL-15 (p=0.078) and TNF-α (p=0.053) were higher; FDR<0.20. We next compared the cytokine signatures in SOTRs with COVID-19 and matched Non-SOT COVID-19 patients. We found higher levels of Eotaxin, GM-CSF, IFN-λ1, IL-13, IL-16, IL-4, IP-10, MCP-1, MCP-2, MCP-4, MDC, MIP-1β, TARC, and TNF-β in SOT COVID-19 patients. These results demonstrate SARS-CoV-2 infection induces distinct cytokine response in SOTRs with COVID-19 compared to Non-SOT COVID-19 patients.

#### Integrated cfDNA and cytokine analysis stratify SOT COVID-19 patients based on disease severity.

**Figure 5.**
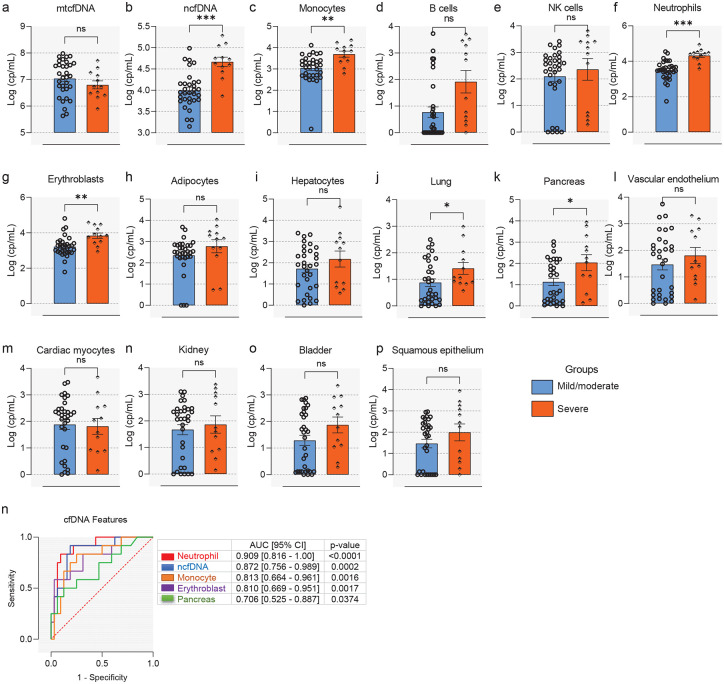
Plasma cfDNA features stratify SOT COVID-19 patients by severity. COVID-19 subjects were grouped based on disease severity as mild/moderate and severe. Comparisons of absolute total mtcfDNA (**a**) ncfDNA and mtcfDNA (**b**) and tissue-specific cfDNA levels derived monocytes (**c**), B cells (**d**), NK cells (**e**), neutrophils (**f**), erythroblasts (**g**), vascular endothelium (**h**), adipocytes (**i**), hepatocytes (**j**), lung (**k**), pancreas (**l**), cardiac myocytes (**m**), kidney (**n**), bladder (**o**), and squamous epithelium (**p**) from SOTRs with mild-moderate (n=12) and severe disease (n=32). (**q**). ROC curve analyses using admission cfDNA profile was performed to identify SOT patients with severe COVID-19. Statistical significance was determined by the Mann–Whitney test. Adjusted p values are reported (for multiple comparison and demographic factors (age, sex, and BMI). Statistical significance was determined by the Mann–Whitney test. A p-value ≤0.05 and FDR≤0.25 was considered significant;*: FDR ≤ 0.25 and p-value ≤ 0.05, **: FDR ≤ 0.1 and p-value ≤ 0.05, ***: FDR ≤ 0.05 and p-value ≤ 0.05, NS: FDR > 0.25 or p-value > 0.05.

We have previously shown cfDNA derived from both hematopoietic and non-hematopoietic tissues were markedly elevated in in Non-SOT patients with poor COVID-19 outcomes^27^. Tissue-specific cfDNA measures showed superior performance to identify patients who eventually develop poor COVID-19 outcomes than previously described conventional inflammatory and organ injury biomarkers^27^. Given the heterogeneous nature of the COVID-19 clinical course, we stratified SOT patients by disease severity to test whether admission cfDNA profile correlated with subsequent clinical trajectory in SOTRs. Based on the maximum WHO scale during COVID-19 hospitalization, subjects were categorized as WHO scale 3–4 assigned for mild/moderate COVID-19 (n = 32, 72.7%) not requiring ICU, or as WHO scale 5–7 assigned for severe COVID-19 (n=12, 27.3%) requiring ICU care^38^. The data showed that the levels of ncfDNA at admission were significantly increased in SOT patients who eventually developed severe disease compared to those patients with mild/moderate COVID-19 (median [IQR] = 47,708 [29,099 – 97,667] cp/mL vs. 8,828 [5,815 – 14,909] cp/mL, p<0.001 and FDR<0.05), but not mtcfDNA (Fig.5a–b). Deconvolution analyses revealed that SOTRs with severe COVID-19 disease displayed distinct tissue injury profiles from those with mild/moderate COVID-19.

They showed significantly increased cfDNA levels derived from monocytes, neutrophils, erythroblast, lung, and pancreas compared to those with mild/moderate disease after adjusting for age, sex, and BMI (p<0.05 and FDR<0.25; Fig.5c, f, g, j and k). Interestingly, the receiver operator characteristic (ROC) curve analysis for the cfDNA features, including neutrophils (AUC = 0.909, 95% confidence interval [CI] = 0.816 – 1.00), ncfDNA (AUC = 0.872, 95% CI = 0.756 – 0.989), monocytes (AUC = 0.813, 95% CI = 0.664 – 0.961), erythroblasts (AUC = 0.810, 95% CI = 0.669 – 0.951) and pancreas (AUC = 0.706, 95% CI = 0.525 – 0.887) showed high to modest performance to identify SOT patients who subsequently develop severe disease/died (Fig.5n, p<0.05).

**Figure 6.**
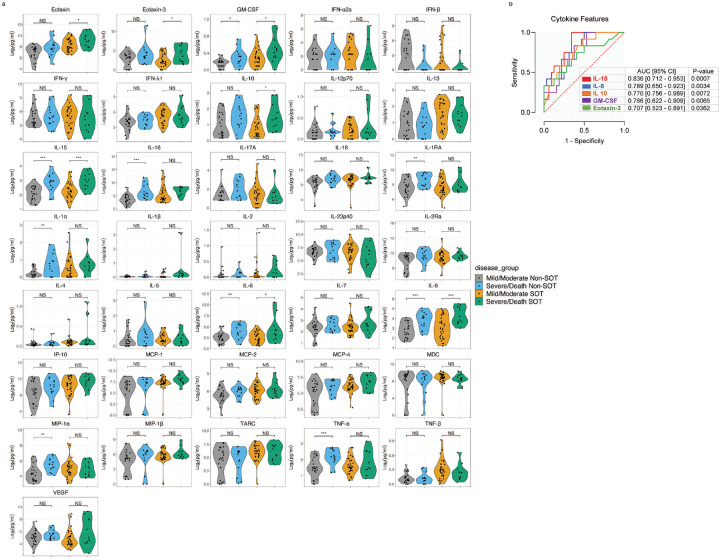
Plasma cytokine levels associated with COVID-19 disease severity in SOTRs. (a). Comparison of plasma cytokine/chemokine levels in mild/moderate (n=32) verses severe SOTRs with COVID-19 (n=12). Cytokine values are reported as picograms per milliliter (pg/mL). (b). ROC curve analyses using admission cytokine profile was performed to identify SOT patients with severe COVID-19. Statistical significance was determined by Mann–Whitney test. Adjusted p values are reported (for multiple comparison and demographic factors (age, sex, and BMI). A p-value ≤0.05 and FDR≤0.25 was considered significant;*: FDR ≤ 0.25 and p-value ≤ 0.05, **: FDR ≤ 0.1 and p-value ≤ 0.05, ***: FDR ≤ 0.05 and p-value ≤ 0.05, NS: FDR > 0.25 or p-value > 0.05.

Next, we investigated whether admission plasma cytokine levels are associated with disease severity (p<0.05 and FDR<0.25; Fig.6a). We observed significantly increased levels of inflammatory cytokines/chemokines such as GM-CSF, IL-15, IL-6 and IL-8 in both SOT and Non-SOT COVID-19 patients who subsquently developed severe disease compared to patients with mild/moderate disease. The levels of Eotaxin, Eotaxin-3 and IL-10 were exclusively elevated in severe SOT COVID-19 whereas IL-16, IL-1RA, IL-1α, MIP-1α and TNF-α, were solely increased in Non-SOT COVID-19 patients with severe disease, as compared to their respective mild/moderate group. Although they did not reach statistical significance, IL-1α (p=0.095 and FDR=0.29), IL-1β (p=0.071 and FDR= 0.26) and MCP-1 (p=0.051 and FDR=0.23) and were higher in SOTRs with severe disease while IFN-α2a (p=0.082 and FDR=0.27) and IFN-β (0.064 and FDR=0.26) were lower. Similarly, Eotaxin (p=0.080), IL-10 (p=0.095), IL-18 (p=0.095) and IL-2Ra (p=0.071) were higher and IFN-β (p=0.094) was lower in Non-SOT COVID-19 patients (FDR<0.26 for all). The ROC curve analysis of each cytokine profile showed modest performance relative to cytokine features to identify SOT patients who develop severe disease, with IL-15, IL-8, IL-10, GM-CSF and Eotaxin-3 yielding significant AUC performance of 0.836 [95% CI = 0.712 – 0.953], 0.789 [95% CI = 0.650 – 0.923], 0.766 [95% CI = 0.622 – 0.909], 0.770 [95% CI = 0.756 – 0.989], and 0.707 [95% CI = 0.523 – 0.891], respectively; Fig.6b. The random forest analysis also identified the most discriminative cfDNA (ncfDNA, neutrophil, monocyte, adipocyte, and erythroblast) and cytokine (IL-15, IL-16, IL-23p40, TNF-α, and IL-6) features at admission for predicting disease severity in SOT patients with high performance (AUC = 0.875 (95% CI: 0.765–0.985) (Suppl.Fig.4a–d).These results demonstrate that integrated cfDNA and cytokine profile may serve as a sensitive approach to identify SOTRs who eventually progressed to severe disease. Furthermore, longitudinal cfDNA and pro-inflammatory cytokine levels remained elevated in SOT COVID-19 patient who eventually died, whereas gradually decreased with time in SOT patients with mild/moderate disease who eventually recovered (Suppl.Fig.5) (see supplementary materials for details).

#### Correlation of cfDNA profiles and cytokine signatures.

**Figure 7.**
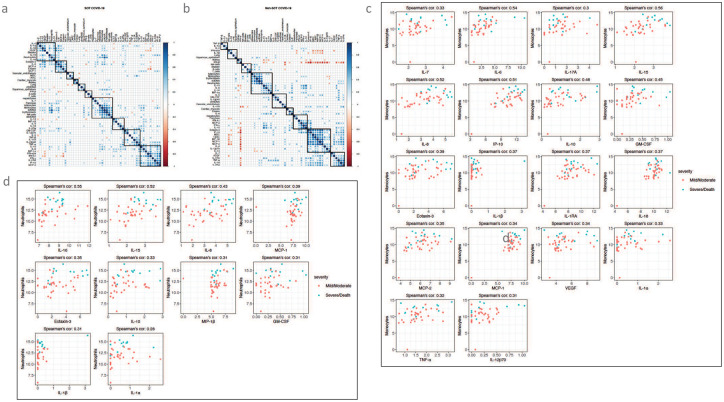
A strong association between circulating cfDNA and inflammatory cytokine/chemokine signatures in SOTRs with COVID-19. Admission plasma cfDNA and cytokine were measured from SOTRs with COVID-19 (n=44) and Non-SOT COVID-19 patients (n=38). Pearson correlation matrix analysis of cfDNA profiles and cytokines/chemokines levels in SOTRs (**a**) with COVID-19 and Non-SOT COVID-19 (**b**). Scatter Plot of the relationship between monocyte derived cfDNA and cytokines in SOT patients with COVID-19 (**c**). Correlation scatter plot plots between neutrophil derived cfDNA and cytokines in SOT patients with COVID-19 (**d**). Adjusted p values are reported (for multiple comparison and demographic factors (age, sex, and BMI). Statistical significance was determined by the Spearman correlations test. A p-value ≤0.05 and FDR≤0.25 was considered significant;*: FDR ≤ 0.25 and p-value ≤ 0.05, **: FDR ≤ 0.1 and p-value ≤ 0.05, ***: FDR ≤ 0.05 and p-value ≤ 0.05, NS: FDR > 0.25 or p-value > 0.05.

To understand the relationship between the host inflammatory responses and tissue injury pattern, we calculated the Spearman’s correlation between all biomarkers pairs (i.e., cfDNA profiles and cytokines) across patients within the SOT COVID-19 and Non-SOT COVID-19 groups. To find biomarkers that are highly correlated with each other, we applied unsupervised clustering to group the biomarkers into ten clusters. We found the size and components of the clusters are different between SOTRs and Non-SOT COVID-19 patients (Fig.7a and b), as well as disease severity subgroups (see supplementary materials for details, Suppl.Fig.6 and 7)). While IL-15 and IL-16 are clustered with neutrophil-derived cfDNA in SOTRs with COVID-19, inflammatory cytokines such as IL-15, IL-6, IL-16, and MIP-1α are clustered with total (ncfDNA) and tissue-specific cfDNA from adipocytes, monocytes, erythroblasts, and neutrophils in Non-SOT COVID-19 patients. The Spearman’s correlation among the biomarkers revealed that global and tissue-specific cfDNA profiles were highly correlated with several cytokine signatures. Specifically, ncfDNA was correlated with cytokines/chemokines such as IL-15, IL-8, Eotaxin-3, IL-1β, IL-10, IL-16, VEGF, IL-6, IL-7, GM-CSF and IL-1α in SOTRs with COVID-19 (Fig 7A, Suppl.Table 2). In Non-SOT patients (Fig7b, Suppl.Table 3), while TNF-α, IL-18 and IL-2Ra were significantly correlated with ncfDNA, MCP-2, GM-CSF, IL-6, IL-10 and IL-8 were marginally correlated. Given the innate immune cells are linked to COVID-19 immunopathogenesis^39^, our analysis revealed monocyte-derived cfDNA showed the strongest correlation with cytokines associated with cytokine release syndrome (CRS)^40^ such as IL-15, IL-6, IL-8, IP-10, IL-10, GM-CSF, Eotaxin-3, IL-1β, IL-1RA, IL-18, MCP-2, MCP-1, VEGF, IL-1α, IL-7, TNF-α, IL-12p70, and IL-17A in SOTRs with COVID-19 (Fig. 7c). Similarly, neutrophil-derived cfDNA was strongly correlated with IL-16, IL-15, IL-8, MCP-1, Eotaxin-3, IL-10, MIP-1β, GM-CSF, and IL-1β (Fig.7d). In Non-SOT COVID-19 patients (Suppl.table 3), we observed 6 (IL-13, IL-10, IFN-γ, IL-12p70, MCP-2, IL-6) and 9 (IL-16, IL-13, TNF-α, IL-6, IL-18, IL-15, MIP-1α, IL-2Ra, IL-8) cytokine correlations with monocyte and neutrophil derived cfDNA, respectively. Additionally, we observed 3 (IL-7, IL-6 and IL-17A) and 1 (IL-17A) correlations between NK cell-derived cfDNA and cytokines; 1 (Eotaxin-3) and 2 (MIP-1α and IL-17A) correlations between B cell-derived cfDNA and cytokines; and 5 (IL-15, TNF-β, IL-7, IL-8, and VEGF) and 1 (IL-13) correlations between erythroblast-derived cfDNA and cytokines levels in SOT and Non-SOT COVID-19 patients, respectively. Among non-hematopoietic tissues, we observed 7 (IL-4, TNF-α, IL-6, IL-10, IP-10, IL-18, MIP-1α) and 2 (IL-16 and IL-6 ) correlations between adipocyte-specific cfDNA and cytokines, 1 (IL-7) and 0 correlations between hepatocyte-specific cfDNA and cytokine, 2 (IL-7 and TARC) and 1 (IL-17A) correlations between and vascular endothelial cell-derived cfDNA and cytokines, 6 (Eotaxin-3, IL-15, IL-8, IL-7, VEGF, IL-16) and 3 (IFN-α2a, MCP-4, IL-1β) correlation between pancreas-specific cfDNA and cytokines, 4 (IL-18, IL-15, IL-1β, IP-10) and 4 (IL-6, GM-CSF, MCP-2, IL-1β) correlation between squamous epithelial cell-derived cfDNA, and 0 and 5 (IL-15, IFN-β, IL-16, IP-10, IL-10) correlation between kidney and cytokines levels in SOTRs and Non-SOT COVID-19 patients, respectively (Suppl.Table 2 and 3). The other cell or tissue types showed no significant correlation with cytokine levels in both groups. These observations provide evidence of distinct interaction of host immune response and cfDNA profiles between SOTRs with COVID-19 and Non-SOT patients.

**Figure 8.**
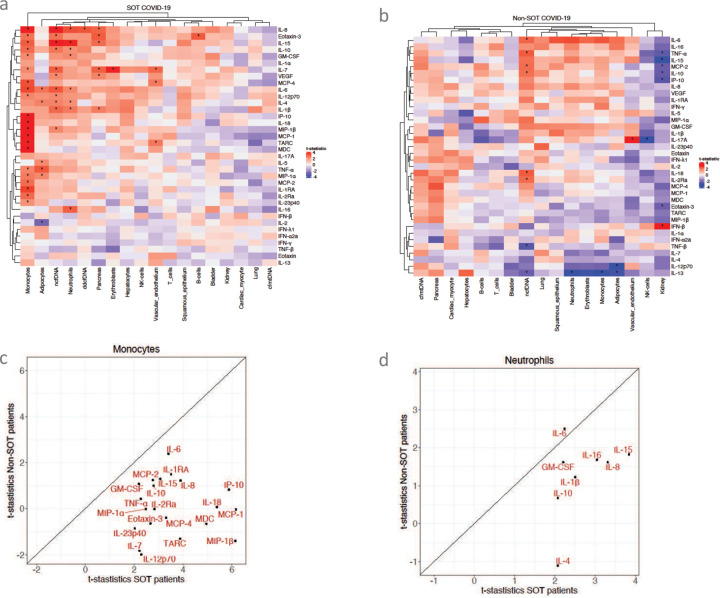
Linear regression analysis reveals an important association between cfDNA and cytokine signatures. Hierarchical Clustering Heatmap of circulating cfDNA and cytokine signatures in SOT (a) and Non-SOT patients COVID-19 (**b**). t-statistics scatterplot plots between monocyte derived cfDNA and cytokines in SOT patients with COVID-19 (**c**). t-statistics scatter plot plots between neutrophil derived cfDNA and cytokines in SOT patients with COVID-19 (**d**). Linear regression analysis between cfDNA features and cytokine profile was conducted. Adjusted p values are reported (for multiple comparison and demographic factors (age, sex, and BMI). A p-value ≤0.05 and FDR≤0.25 was considered significant;*: FDR ≤ 0.25 and p-value ≤ 0.05, **: FDR ≤ 0.1 and p-value ≤ 0.05, ***: FDR ≤ 0.05 and p-value ≤ 0.05, NS: FDR > 0.25 or p-value > 0.05.

To further explore the potential association between circulating cytokine levels and the extent of global and tissue-specific injury profiles, we applied a linear regression analysis to test the association between each cytokine and each cfDNA feature (see methods) (Fig.8a and b, suppl.Fig.8, suppl.Table 4 and 5). The t-statistics from the coefficient of the linear regression are shown in Figure 8a and b. Significant associations (FDR < 0.25) are marked with stars. The results showed that cytokines/chemokines such as IL-8, IL-15, IL-6, IL-7, IL-1β, Eotaxin-3, IL-10, IL-4, MIP-1β, IL-12p70 and VEGF were associated with global tissue injury profile, i.e., ncfDNA, in SOTRs with COVID-19, whereas TNF-β, TNF-α, IL-18, IL-6, IL-13, IL-2Ra, MCP-2, and IL-10 were associated with global tissue injury in Non-SOT patients. More importantly, monocyte-derived cfDNA was not clustered with other cfDNA parameters and associated with most of the proinflammatory cytokines and chemokines in SOTRs with COVID-19 (Fig8a, Fig.8c). The strongest association was found with MCP-1 (t-statistics= 6.2, p=2.29E-07), MIP-1β (t-statistics= 6.1, p=2.46E-07), IP-10 (t-statistics= 5.9, p=5.97E-07), IL-18 (t-statistics= 5.4, p=2.96E-06), MDC (t-statistics= 5.0, p=1.23E-05), IL-8 (t-statistics= 3.9, p=0.0003), TARC (t-statistics= 3.9, p=0.0004), IL-1RA (t-statistics= 3.5, p=0.001), IL-6 (t-statistics= 3.4, p=0.0015), MCP-4 (t-statistics= 3.3, p=0.0019), IL-15 (t-statistics= 3.1, p=0.004), IL-2Ra (t-statistics= 2.8, p=0.007), IL-10 (t-statistics= 2.8, p=0.008), MCP-2 (t-statistics= 2.8, p=0.008). Levels of Eotaxin-3, MIP-1α, IL-12p70, TNF-α, IL-7, GM-CSF, and IL-23p40 were also significantly associated with monocyte-derived cfDNA in SOTRs with COVID-19 (p<0.05 and FDR<0.25). Conversely, monocyte-derived cfDNA was co-clustered with cfDNA derived from neutrophils, erythroblast, adipocytes, and NK cells and negatively associated with IL-13 in Non-SOT patients (Fig.8b). Likewise, neutrophil-derived cfDNA was associated with cytokines and chemokines such as IL-15, IL-8, IL, IL-1β, IL-6, GM-CSF and IL-10 in SOT patients (Fig.8d), whereas in Non-SOT patients neutrophil-derived cfDNA was negatively associated with IL-13. Additionally, the following association has been observed in SOTRs with COVID-19: adipocyte-derived cfDNA with TNF-α, IL-2, IL-6, IL-4, MIP-1α, and IL-5; vascular endothelium-derived specific cfDNA with IL-7, TARC, MCP-4; pancreas-derived cfDNA with Eotaxin-3, IL-7, IL-8, IL-15, IL-1β, and VEGF; and ddcfDNA with IL-8. On the other hand, Non-SOT COVID-19 patients showed the following associations: NK cells with IL-17A; Adipocyte with IL-12p70 and IL-13; and Kidney with IFN-β (positively) and [IL-15, IP-10, TNF-α, IL-10, MCP-2, and Eotaxin-3; negatively) has been significantly correlated in non-transpant COVID-19 patients. Collectively, these findings suggested that monocyte-macrophage cell lineages and neutrophils are the major players of COVID-19 immunopathology.

#### COVID-19 shows distinct tissue injury profiles compared to other respiratory viruses.

**Figure 9.**
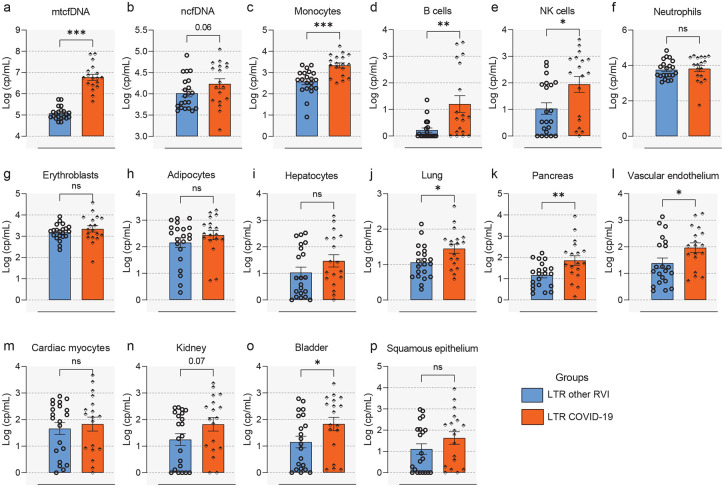
High plasma cfDNA levels in lung transplant recipients with COVID-19 compared to other respiratory viruses. Comparisons of absolute total mtcfDNA (**a**) ncfDNA and mtcfDNA (**b**) and tissue-specific cfDNA levels derived monocytes (**c**), B cells (**d**), NK cells (**e**), neutrophils (**f**), erythroblasts (**g**), vascular endothelium (**h**), adipocytes (**i**), hepatocytes (**j**), lung (**k**), pancreas (**l**), cardiac myocytes (**m**), kidney (**n**), bladder (**o**), and squamous epithelium (**p**) among lung transplant recipients with COVID-19 (n=18) and other respiratory viral infections (other RVIs) (n=21). Statistical significance was determined by the Mann–Whitney test. Adjusted p values are reported (for multiple comparison and demographic factors (age, sex & BMI). A p-value ≤0.05 and FDR≤0.25 was considered statistically significant;*: FDR ≤ 0.25 and p-value ≤ 0.05, **: FDR ≤ 0.1 and p-value ≤ 0.05, ***: FDR ≤ 0.05 and p-value ≤ 0.05, NS: FDR > 0.25 or p-value > 0.05.

COVID-19, like other RVIs such as influenza A and B, respiratory syncytial virus, parainfluenza, and rhinovirus are associated with significant morbidity and mortality in SOTRs, particularly among LTRs^41–43^. However, the extent of tissue injury of SARS-CoV-2 compared to other RVIs has not been studied previously. We compared the cfDNA profile in LTRs with SARS-CoV-2 (n=21) to LTRs with other RVIs (n=21). The median mtcfDNA level was higher in LTRs with COVID-19 (median [IQR] = 5,580,254 [2,435,676 – 13,148,455] cp/mL) than for LTRs with other RVIs (median [IQR] = 113,985 [75,197 – 156,411] cp/mL) (Fig.9a, p<0.0001) matched for disease severity using WHO Ordinal Scale. The ncfDNA level was marginally higher in LTRs with COVID-19 (8,911 [4,603 – 19,881] cp/mL vs 16,483 [6,789 – 46,123] cp/mL, Fig.9b, p=0.0646) as compared with LTRs with other RVIs. Interestingly, the tissue-specific cfDNA levels were different, with LTRs with COVID-19 showing increased levels of ncfDNA from monocytes, B cells, NK cells (Fig.9c–e), vascular endothelium (Fig.9j), pancreas (Fig.9k), bladder (Fig.9i), and lung (Fig.9o) in their plasma compared to other RVIs; p<0.05 for all). There was no significant difference in other cell or tissue types.

#### Allograft injury is elevated in COVID-19 SOTRs.

**Figure 10.**
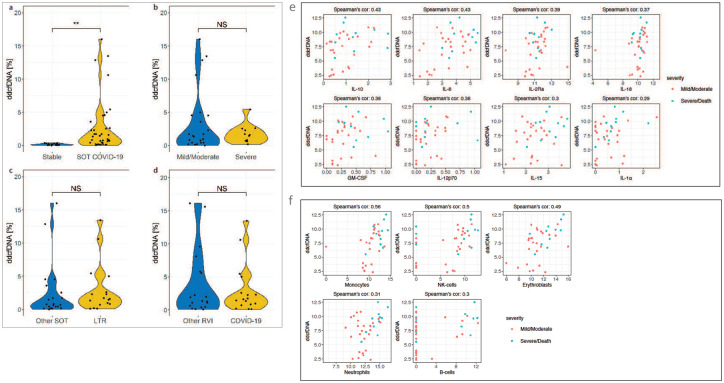
Elevated ddcfDNA level in SOTRs with COVID-19. (a) comparison of ddcfDNA level between stable transplant recipients (n=18) and SOTRs with COVID-19 (n=39). (a) comparison of ddcfDNA level between stable controls (n=21) and SOTRs with COVID-19 (n=44). (b) comparison of ddcfDNA in SOT patients with mild/moderate (n=28) and severe COVID-19 disease (n=12). (c) comparison of ddcfDNA level in lung transplant recipients with COVID-19 (LTR) (n=17) and other SOT types (n=22). (c) comparison of ddcfDNA level in lung transplant recipients with COVID-19 (LTR) (n=17) and other RVIs (n=19). (d) Correlation scatter plot plots between ddcfDNA and cytokines in SOT patients with COVID-19. (e) Correlation scatter plot plots between ddcfDNA level and total and tissue-specific cfDNA profiles in SOT patients with COVID-19. Statistical significance was determined by Mann–Whitney test and Spearman correlations test. Adjusted p values are reported (for multiple comparison and demographic factors (age, sex, and BMI). A p-value ≤0.05 and FDR≤0.25 was considered statistically significant;*: FDR ≤ 0.25 and p-value ≤ 0.05, **: FDR ≤ 0.1 and p-value ≤ 0.05, ***: FDR ≤ 0.05 and p-value ≤ 0.05, NS: FDR > 0.25 or p-value > 0.05.

We demonstrated previously that plasma ddcfDNA fraction (%) is a sensitive biomarker for allograft injury, and detects allograft rejection and predict long-term outcomes in SOTRs^22,23^. The ddcfDNA released from transplanted organs into the recipient circulation is not specific to allograft rejection; elevations have been observed during viral infections and other pathological conditions^44^. Here we explored whether ddcfDNA could detect allograft injury in SOTRs infected with SARSCOV2. We found that %dd-cfDNA was significantly higher (median [IQR] = 1.34% [0.41–2.59%]) in SOTRs with COVID-19 than stable transplant controls (median [IQR] = 0.13% [0.067–0.25%]) (Fig.10a, p< 0.001). 79.5% of the COVID-19 transplant patients showed higher ddcfDNA levels than median stable transplant values, including 83% of lung, 66.7% of kidney, 66.7% of the heart, all the liver, and one multiorgan transplant patient. Although it didn’t reach statistical significance, the fraction of ddcfDNA tended to be higher in patients who develop severe disease compared to mild cases (median[IQR]: 1.52% [0.69–2.46] vs 0.97% [0.19 – 4.5] (Fig.10b). Previous studies showed the ddcfDNA fraction calculation is influenced by the total recipient-derived cfDNA concentration and COVID-19 patients release excessive total cfDNA^45^; this may mask the intensity of allograft injury. The ddcfDNA test used in this study does not incorporate absolute dd-cfDNA levels and consideration of total cfDNA levels in assessing allograft injury may show a more exuberant allograft injury with COVID-19. Although ddcfDNA level didn’t show significant difference, the median %ddcfDNA tends to higher in LTRs with COVID-19 (median [IQR] =1.6 % [0.76 – 3.79]) as compared with other SOT types with COVID-19 (median [IQR] = 0.71% [0.19 – 2.79] or to LTRs with other RVIs (median [IQR] =1.37% [0.42 – 5.8]; Fig.10c and d, p>0.05 for both comparisons. An additional finding from this study is ddcfDNA was strongly correlated with plasma cytokines/chemokine such as IL-10 (r=0.43), IL-8 (r=0.43), IL-2Ra (r=0.39), IL-18 (r=0.37), GM-CSF (r=0.36) and IL-12p70 (r=0.35), and marginally correlated with levels of IL-15 (r=0.30, p=0.07), IL-1α (r=0.29, p=0.08); (Fig.10e, FDR<0.25 for all). ddcfDNA were also correlated with cfDNA levels derived from NK-cells (r=0.54), monocytes (r=0.50), erythroblast (r=0.49), and marginally correlated with neutrophil (p=0.069) and B-cell (p=0.07); (Fig.10f, FDR<0.25 for all. We observe that COVID-19 triggers allograft injury, even for allografts distant from the primary site of SARS-COV-2 infection. The correlation of allograft injury to cytokine levels suggests that dysregulated cytokine response and immune cells may potentially contribute to allograft injury.

## 3. Discussion

Based on prior experience with other respiratory viral infections, SOTRs are at higher risk of severe complications and deaths^41–43^. Early identification of tissue injury, including allograft injury, as well as inflammatory response, may further illucidate disease pathogenesis, guide risk stratification, inform therapeutic options to improve long-term outcomes of SOTRs. This study performed the first integrated cfDNA and cytokine analysis to elucidate the tissue injury patterns and immune response in SOTRs with COVID-19, including a direct comparison with immunocompetent patients and SOTRs with other respiratory viruses. We leveraged a biomarker of the tissue injury –cell-free DNA– to measure allograft injury as ddcfDNA and injury from different tissue types using cell/tissue-specific DNA methylomic signatures. The tissue-specific cfDNA measures correlated with known markers of tissue injury, indicating that this cfDNA approach reliably measures tissue injury. In accordance with the known systemic clinical manifestations of COVID-19^46^, we found evidence of tissue injury from diverse cell or tissue types, including hematopoietic cells, vascular endothelium, adipocyte, pancreas, hepatocyte, kidney, heart and lung. cfDNA from these tissue types also showed high performance to identify patients who subsequently developed severe disease/died. Moreover, we analyzed immune responses of SOTRs with COVID-19 using multiplex cytokine profiling in the bloodstream of patients and correlated them with cfDNA profiles. Our analysis of circulating cytokine and chemokine signatures revealed distinct patterns in SOTRs with COVID-19 and the majority of the cytokines were correlated with monocyte-derived cfDNA. Additionally, IFN-λ1, a type III interferon, was markedly elevated in SOTRs with COVID-19 compared to Non-SOT COVID-19 patients. This was an unexpected finding as IFN-λ1 and type I interferons are thought to be suppressed in SARS-CoV-2 infections^47^. Moreover, IFN-λ1 is actually higher in influenza infection than in SARS-CoV-2^31^.

COVID-19 is caused by a complex interplay between direct SARS-CoV-2 infection and subsequent inflammatory responses^48,49^. However, there are conflicting reports on the impact of immunosuppressive drugs on the severity of COVID-19 in transplant recipients^12,50–53^. Most of these studies are based on traditional inflammatory and organ injury markers, and definitive diagnoses with these conventional biomarkers are challenging due to their low sensitivity and predictive performance^54^. Circulating cfDNA, which carries genetic and epigenetic information from tissues-of-origin, is a sensitive noninvasive biomarker in multiple clinical settings, such as cancer^55^, infectious diseases,^27,28^ and allograft rejection^22^. The cfDNA measures from various tissue types correlated with disease clinical trajectory and outcome and have superior predictive performance over established laboratory markers^27^. This study performed the first integrated cfDNA and cytokine analysis to elucidate the tissue injury patterns and immune response in SOTRs with COVID-19, including a direct comparison with immunocompetent patients. Our data show that total plasma cell-free nuclear and mitochondrial DNA, a measure of global cellular death and tissue injury, were markedly elevated in SOTRs in contrast to stable transplant patients and healthy controls. Interestingly, our result also demonstrated plasma ncfDNA, was significantly lower in SOTRs in contrast to Non-SOT patients. Importantly, our analysis indicated that ncfDNA levels were significantly higher in SOT COVID-19 patients who eventually developed severe disease or died, suggesting it is a sensitive marker of global tissue injury. However, the mtcfDNA was an imperfect predictor of disease progression/outcome, and our findings disagree with a recent study that reported mtcfDNA as a predictive marker of poor COVID-19 outcomes in Non-SOT settings^56^.

In accordance with previous reports^31,37^, our plasma cytokine profiling revealed ongoing alteration of circulating cytokine levels in both SOTRs and Non-SOT COVID-19 patients. We have identified 11 cytokine/chemokine differences in both SOT and Non-SOT COVID-19 patients as compared to healthy controls, including cytokines with higher (IFN-λ1, MCP-2, IL-6, IL-1RA, IFN-γ) or lower (Eotaxin-3, MDC, TARC, IL-13, MCP-4, IL-12p70) levels. While the levels of IP-10, IL-15 and IL-18 were exclusively increased in SOTRs with COVID-19, Non-SOT patients with COVID-19 showed solely higher levels of IFN-β, IL-2, IL-10, GM-CSF and low levels of Eotaxin, MCP-1, MIP-1β, IL-4, IL-5. Additionally, high IFN-λ1, GM-CSF, MCP-2, TNF-β, IL-16 and low eotaxin-3 were seen in SOTRs with COVID-19 as compared to Non-SOT patients. The levels of inflammatory cytokines/chemokines such as IL-8, IL-15, IL-10, GM-CSF, IL-6, Eotaxin and Eotaxin-3 were increased in the bloodstream of SOT COVID-19 patients who eventually developed severe disease/died but showed a modest discriminatory performance to distinguish mild/moderate to severe disease. Although an exacerbated inflammatory response is a leading cause of severe COVID-19 immunopathology; the driving factors that lead to excessive inflammation in COVID-19 patients remain poorly defined. The correlation analysis in our study revealed a distinct correlation between cfDNA and cytokine correlations in SOTRs with COVID-19.

Prior studies also reported a bidirectional relationship between cfDNA level and inflammation because cfDNA can trigger a deleterious proinflammatory response. In turn, the inflammation causes excessive cfDNA release, enhancing tissue injury^35^. We have also shown the excessive cfDNA released in COVID-19 patients caused the overproduction of mitochondrial reactive oxygen species, increasing tissue injury^27^. Moreover, elevated plasma cfDNA levels induce coagulation activation and impair fibrinolysis^57,58^, thereby contributing to microvascular thrombosis. Remarkably, IL-13, the prototypical Th2 cytokine, was associated with protection against multiple-tissue injury in Non-SOT patients. Nonetheless, these findings suggest that simultaneous immunostimulation and immunosuppression occur in SOT patients that may have a protective or pathologic effect.

Although the lung is the primary target organ for SARS-CoV-2, multiple cells, tissue, or organ types can be involved due to broad SARS-CoV-2 cellular tropism^59^ and subsequent inflammatory responses^48^. Our deconvolution results indicated that hematopoietic cells, including granulocytes, erythrocyte progenitors, monocytes, NK cells and B cells, are the predominant source of circulating cfDNA levels in SOTRs with COVID-19. Elevated cfDNA derived from these cell types may be associated with high cellular death/injury from both direct infection with SARS-CoV-2 and indirect effects of systemic inflammation and turnover^60,61^. Neutrophils and monocytes are essential components of innate immunity and appear to play a dominant role in COVID-19 pathogenesis^39^. Our observation also showed neutrophils to be the leading contributor to an elevated cfDNA level in SOTRs with COVID-19. Additionally, neutrophil-derived cfDNA appeared to be an excellent predictor of progression to severe disease/death. Neutrophils expel cfDNA into the extracellular environment during neutrophil extracellular traps (NETs) formation. The excessive NET formation has been noted in COVID-19 patients and is linked to disease severity^62–64^. Our result also indicated a strong positive correlation between neutrophil-derived cfDNA and D-dimer (a degradation product of fibrin), implicating their role in immunothrombosis. Eotaxin-3, a potent chemoattractant of eosinophils, was markedly decreased in COVID-19 patients, with more elevation in SOTRs with COVID-19. In line with this, admission blood sample analysis revealed eosinopenia in COVID-19 patients who eventually progressed to severe disease/died. Monocytes, a precursor of macrophages, play a causal role in severe COVID-19 immunopathology by driving cytokine storm^39^. We find high monocyte-derived cfDNA in SOTRs with COVID-19 that correlate with subsequent disease severity. Interestingly, we find strong correlations between monocyte-derived cfDNA and proinflammatory cytokines such as TNF-α, MCP-1, IL-18, and IL-6 in SOTRs as compared to Non-SOT patients. Moreover, our hierarchical linear regression analysis identified monocytes were strongly associated with the majority of inflammatory cytokines in SOT COVID-19 patients. Particularly, our multivariate linear regression analysis showed cytokines/chemokines such as IL-6, IL-8, MIP-1α, GM-CSF, MCP-4, IL-18, TNF-α, IL-15, IP-10, MDC, IL-1RA, MCP-1, and MIP-1β as effectors of monocyte injury in SOTRs. Most strikingly, the correlation and linear regression analysis between monocyte-derived cfDNA and cytokines were stronger in SOTRs with severe disease. These findings highlight monocytes as the major contributor to severe immunopathology in transplant patients COVID-19. The current immunosuppressive regimens (e.g., mycophenolate mofetil and calcineurin inhibitors) are not thought to target the monocyte cell lineages, and the effects on cytokine production are limited^65^.

COVID-19 patients also exhibited erythropoiesis aberrations, with increasing erythroid progenitors in the bloodstream of patients with severe disease^66^. Our data corroborated a role for altered erythropoiesis in COVID-19 with elevated circulating cfDNA originating from erythroid progenitors, especially in severe disease. Another feature of COVID-19 is lymphopenia in the peripheral blood (T, B, and NK cells), indicating impairment of adaptive immunity^67^. Absolute lymphocyte count was also low in SOT COVID-19 patients and our methylome analysis corroborated these findings with low/undetectable cfDNA levels from CD4^+^ T cells and CD8^+^ T cells. This might be due to the nature of SARS-CoV-2 infection or the immunosuppressive treatments. A recent study also showed extensive NK cell activation, tissue trafficking, and turnover in COVID-19 patients correlated with subsequent disease progression^60^. Consistent with this study, we found high cfDNA derived from NK cells at admission to be associated with subsequent disease severity. Our work and that of others demonstrate significant injury from non-hematopoietic tissue types in Non-SOT COVID-19 patients^27,28^. Similarly, we found considerable injury from vascular endothelium, adipocytes, liver, pancreas, kidney, heart, and lung in SOTRs with COVID-19 compared to healthy controls and to stable transplant patients. These results suggest the involvement of multiple cell, tissue or organ types in COVID-19 pathogenesis and the results are consistent with clinical presentations of patients with COVID-19^29^. While we could not find statistically significant differences for the majority of tissue-specific cfDNA levels in transplant versus Non-SOT patients with COVID-19, B cells and cardiac myocytes-derived cfDNA levels were high in Non-SOT COVID-19 patients.

The dd-cfDNA released from the transplanted organ is not specific to rejection, and elevations are also observed during viral infections without concomitant allograft rejection^44^. Viral infections affect allograft function by both direct tissue damage and immunologically induced injury. In this study, we found ddcfDNA was significantly elevated in SOTRs with COVID-19 and strikingly correlated with cytokines/chemokines (IL-10, IL-8, IL-2Ra, IL-18, GM-CSF and IL-12p70) associated with CRS. This suggests the allograft undergoes significant injury following SARS-CoV-2 infection and the subsequent inflammatory response and highlights therapeutic targets to prevent allograft injury. Additionally, the increased circulating cfDNA levels may act as DAMP to activate proinflammatory response through a dsDNA pattern recognition receptor and cause allograft dysfunction^68,69^. Aside from the lung, injury has been detected in transplanted organs distant from the primary site of the infection, such as kidney, liver, and heart. More recently, two case reports showed an increase ddcfDNA in heart and kidney transplant patients with COVID-19^70,71^. However, total and tissue-specific cfDNA profiles were not different among SOTRs. Similarly, a multicenter clinical study also reported type of transplant was not associated with COVID-19 severity^5^. This supports that COVID-19 is a systemic disease.

Another interesting observation from this study was extensive tissue injury in LTRs with COVID-19 as compared with other RVIs. Indeed, histologic analysis of pulmonary vessels in patients with COVID-19 also showed widespread vascular endothelialitis, thrombosis, and angiogenesis, which distinguishes COVID-19 from severe influenza or respiratory syncytial virus infection^46^. Consistently, we found significantly increased cfDNA levels from the vascular endothelium and immune cells. Although no significant difference was found in allograft injury, measured as %ddcfDNA between LTRs infected with COVID-19 and those with influenza and/or RSV, the cfDNA methylation analysis revealed high levels of lung-derived cfDNA in LTRs with COVID-19. A plausible explanation for this difference is that the %ddcfDNA calculation is influenced by the total recipient-derived cfDNA concentration^45^, and SOTR COVID-19 patients release excessive total cfDNA. Thus, it may mask the intensity of allograft injury in LTRs with COVID-19 infection. The ddcfDNA test does not incorporate absolute dd-cfDNA levels^72^, and consideration of total recipient-derived cfDNA levels in assessing allograft injury may resolve this discrepancy.

There are some limitations that must be noted for this study. First, transplant patients take a combination of immunosuppressive drugs and the net potential effect of each drug type on cfDNA/cytokine level could vary, and these may contribute to intra- and interindividual variability. Second, human tissues are complex, containing a mixture of different cell types, and the deconvolution methods are restricted to predominant cells in each tissue type included in the reference panel^19^. Thus, the result does not account for all cell types and may not be representative of the tissue profile or may have missed clinically relevant information from other cell types of the tissue. In addition, the cause of cellular injury or destruction that results in cfDNA release from diverse tissue types of the body is not clear. Third, the sample size is small due to relatively few cases of transplant patients infected with SARS-CoV-2, and the under-representation of heart transplant recipients in our cohort. Fourth, SARS-COV-2 variants were not specified in these patients. The tissue injury profiles described here may be different with different SARS-COV-2 variants. Despite these limitations, integrated cfDNA and cytokine analysis captured clinically relevant information in both transplant and Non-SOT populations with COVID-19.

In summary, we show that cfDNA is a comprehensive molecular biomarker to map injury from diverse tissue types early in the course of COVID-19 and predicts subsequent clinical trajectory. We have identified distinct cytokine features in transplant versus Non-SOT COVID-19 patients. Additionally, we found significantly elevated allograft injury in SOTRs with COVID-19. In SOTRs, monocyte-derived cfDNA and ddcfDNA were strongly associated with proinflammatory cytokines involved in CRS. Measuring plasma cfDNA early in the course of COVID-19 may help high-risk patients and guide early initiation of appropriate therapies. Further research is needed to further explore the biological interaction between cfDNA and cytokines. These studies may also investigate the clinical utility of early cfDNA measures to guide treatment plan.

## 4. Materials and Methods

### 4.1. Study Subjects and Setting

A prospective cohort study was conducted at Johns Hopkins Hospital (Clinical Trials.gov identifier = NCT04496466) and University of Maryland Medical Center from April 21, 2020 to September 08, 2021 and include consecutive hospitalized solid organ transplant recipients (n=44) with a confirmed diagnosis of COVID-19 by positive RT-PCR assay for SARS-CoV-2 RNA on a nasopharyngeal swab. All study subjects provided informed consent for blood sample collection at admission and for collection of clinical data, including demographic data, comorbidities, laboratory test results, medications, and other clinical parameters. A subset of patients (n=6) underwent serial blood sample collection during their hospital stay. The specimens utilized for this publication were part of the Johns Hopkins Biospecimen Repository, which is based on the contribution of many patients, research teams, and clinicians. The maximum World Health Organization (WHO) disease severity score reached at any time during the COVID-19 hospitalization was used to categorize patients as mild/moderate (WHO scale 3, 4) and severe disease (WHO scale 5 – 7)^38^. The study also collected blood plasma samples from WHO-scale-matched Non-SOT COVID-19 patients (n=40), lung transplant recipients (LTRs) infected with other respiratory virus infections (other RVIs) (n=18, 21 episodes), stable transplant recipients without acute rejection or infection (n=21) and healthy controls (n=30) to serve as a comparator group. In our cohort, there were no SOTRs with COVID-19 on extracorporeal membrane oxygenation (ECMO) and we excluded the Non-SOT COVID-19 patients receiving ECMO support at any time course of the disease from the analysis. Patients without admission plasma samples were also excluded from the analysis. The study was approved by the Institutional Review Boards of the Johns Hopkins University School of Medicine and University of Maryland Medical Center.

### 4.2. Plasma sample processing and cfDNA isolation

Plasma samples were isolated from whole blood collected in Cell-Free DNA BCT® (Streck, La Vista, NE) or EDTA (BD) tubes by centrifugation at 1600g for 10 min at 4°C, aliquoted and stored immediately at −80°C. The aliquoted plasma was thawed at room temperature and centrifuged at 16000g for 5 min at 4 °C to remove residual debris. cfDNA was extracted from 1 mL of plasma by QIAsymphony circulating DNA kit (QIAGEN). The plasma samples were spiked with 0.142 ng/mL unmethylated lambda phage DNA (Promega), which was fragmented to 160 bp, to measure the efficiencies of cfDNA extraction and bisulfite conversion for genome-wide methylation sequencing. The isolated cfDNA were eluted into 60 μL elution buffer and frozen at −20 °C until ready for further use.

### 4.3. cfDNA quantification

#### 4.3.1. Real-time quantitative PCR (qPCR)

The cfDNA was quantified by qPCR for human Alu repeats (Alu115 and Alu247) to check the integrity (ALU247/ALU115). Concurrent qPCR of lamda DNA was used to measure extraction efficiency. Briefly, a 10μl PCR mixture containing 2μL cfDNA template (1:10 diluted), 5μL SYBR Green SuperMix (Bio-Rad), 2μl nuclease-free water, and 1μL primer pair were prepared in triplicate for each amplicon. The PCR reaction mixture was run on QuantStudio 3 (Applied Biosystems) as follows: initial denaturation at 95 °C for 5 min, followed by 35 cycles of 95 °C for 15 s and annealing at 60 °C for 1 min using QuantStudio^™^ design and analysis software. The primer sequences were as follows: forward, 5′-CCTGAGGTCAGGAGTTCGAG-3′ and reverse, 5′-CCCGAGTAGCTGGGATTACA-3′ for Alu-115; forward, 5′-GTGGCTCACGCCTGTAATC-3′ and reverse, 5′-CAGGCTGGAGTGCAGTGG-3′ for ALU247; and forward, 5′-GACCTCTATGCCAACACAGT-3′ and reverse, 5′-AGTACTTGCGCTCAGGAGGA-3′ for λ DNA. The concentrations for each amplicon were calculated using a standard curve generated from 10-fold serially diluted human genomic DNA (Promega), which was fragmented to 160 bp. The concentration of short fragments (Alu115) reflects total cfDNA level (ng/ml), whereas the ratio of shorter fragments (Alu115) to longer fragments (Alu247) was used to estimate DNA integrity. Samples with an integrity score > 0.8 were eliminated since these are considered to be contaminated with high molecular weight nuclear DNA. The extraction efficiency was calculated as the ratio of input and output of the lambda DNA, which is spiked into plasma samples prior to cfDNA isolation and measured after cfDNA extraction.

#### 4.3.2. Droplet digital PCR (ddPCR)

Absolute copy numbers of ncfDNA and mtcfDNA were quantified on QX 200 Droplet Digital PCR (ddPCR) system, using primers and probes targeting eukaryotic translation initiation factor 2C1 (EIF2C1) found on the nuclear genome and NADH dehydrogenase 1 (ND1) found on the mitochondrial genome, respectively. The probes targeting EIF2C1 were labeled with HEX (BioRad, #10031245) and ND1 probes with FAM (BioRad, #10042960). The ddPCR was carried out in triplicate of 22 μl reaction volume containing 11 μL 2XddPCR Supermix for Probes (No dUTPs), 4 μL of cfDNA (1:10 diluted), and 0.55 μL of each 20X primer/probe sets and 5.9 μL of nuclease-free water, followed by partitioning into ~17,000 droplets. The generated droplets were subjected to PCR amplification at 95 °C for 10 min, followed by 40 cycles of 15 s at 95 °C and 1 min at 60 °C with ramp rate set to 2.5 °C/s between temperatures. After amplification, the data were acquired using QuantaSoft^™^ software on QX200 droplet reader and analyzed using QuantaSoft^™^ Analysis Pro software. Absolute copy number of ncfDNA and mtcfDNA were then normalized by plasma volume and extraction efficiency as follows.


$$\text{Copies}/\text{mL plasma}=\left(\text{A copies}/\mu \text{L}\right)\times \left[\frac{22\mu \text{L of ddPCR rxn}}{\text{B}}\times \text{DF}\right]\times \left[\frac{\text{elution volume}}{\text{C mL of plasma used}\times \text{EF}}\right]$$

Where **A** is copies of ncfDNA or mtcfDNA per [μL] as calculated by Quanta soft software, **B** is the diluted cfDNA volume [μL] used for ddPCR reaction mixture, DF is the dilution factor of the cfDNA sample used for ddPCR reaction mixture, **C** the plasma volume [mL] used for isolation of cfDNA and EF the extraction efficiency as calculated by dividing lambda DNA detected per [μL] over spiked lambda DNA.

#### 4.3.3. Donor-derived cell-free DNA

Five to 10 ng of isolated cfDNA were subjected to next-generation sequencing (NGS), targeting 405 SNPs, to precisely quantify dd-cfDNA with a range of 0.12% – 16%, without the need for donor and recipient genotyping^73^.

### 4.4. Library preparation and sequencing

Size distribution of the cfDNA was determined using Cell-free DNA ScreenTape assay on Agilent 4150 TapeStation System according to the manufacturer’s instructions to assure the absence of contamination with genomic DNA. An input of 5 – 50 ng of isolated cfDNA, depending on the availability, was used to perform bisulfite conversion using EZ DNA methylation-Gold kit (Zymo Research) as per the manufacturer’s recommendation. Libraries were prepared using the Accel-NGS Methyl-Seq DNA Library Kit with Unique Dual Indexing (Swift Biosciences) for whole-genome bisulfite sequencing according to the manufacturer’s instructions. The quality of the constructed cfDNA library was visualized using a high-sensitivity D1000 ScreenTape and quantified using the Quant-iT PicoGreen dsDNA Assay kit (Life Technologies). The DNA libraries were then normalized in equimolar concentrations and were subjected for ~200 M reads by 2X100bp, paired-end DNA sequencing on the Illumina NovaSeq 6000.

### 4.5. cfDNA sequence analysis

The raw sequence reads were quality checked with FastQC^74^ and trimmed using trim_galore^75^. Adapter sequences and 10 base pairs from both pair-end reads were (paired-end reads >50bp were retained). The paired-end sequence reads were subsequently aligned to a C-to-T converted human reference genome (hg38 assembly) using Bismark^76^. Duplicate sequence reads were also removed and post alignment quality control checked using Bismark. The Bismark methylation extractor routine determines cytosine methylation status and extracts all CpGs in each sample. We built a custom bisulfite-sequencing analytic workflow for analyzing and visualizing the cfDNA methylome data using a collection of tools from bsseq^77^. A reference meth_atlas algorithm was used to deconvolute the composition of plasma cfDNA. The reference meth-atlas algorithm is constructed using CpG methylation dataset of 25 human cell or tissue types. The method covers in silico simulations of *in vitro* mixes of known proportion of cfDNA derived from different tissue types. The cfDNA methylation levels of CpG sites represented as a linear combination of those of 25 cell or tissues types and effectively identify and determine the relative contributions of different cell or tissue types to the plasma cfDNAs with ~4,000 CpGs^19^. We filtered CpGs with at least 5x coverage in individual samples for the deconvolution analysis for each cfDNA sample. The deconvolution algorithm scripts provided at https://github.com/nloyfer/meth_atlas and the methylation analysis scripts at https://github.com/seifudd/cfMethylome. Absolute copies of cell, tissue or organ-specific cfDNA per mL plasma (cp/mL) were calculated by multiplying the relative estimated proportions of each cell, tissue or organ type by absolute ncfDNA level (cp/mL).

### 4.6. Cytokine measurement

Cytokines and chemokines (IFN-α2a, IFN-β, IL-18, IL-1RA, IFN-λ1, IL-2Ra, MCP- 2, GM-CSF, IL-23p40, IL-15, IL-16, IL-17A, IL-1α, IL-5, IL-7, TNF-β, VEGF, Eotaxin, Eotaxin-3, IP-10, MCP-1, MCP-4, MDC, MIP-1α, MIP-1β, TARC, IFN-γ, IL-10, IL-12p70, IL-13, IL-1β, IL-2, IL-4, IL-6, IL-8, TNF-α) were measured in plasma using a custom multiplex kit from Meso Scale Diagnostics (MSD, Rockville, MD) according to the manufacture’s protocol. Raw data were acquired using a MESO QuickPlex SQ 120. Each sample was measured in duplicate. If an analyte signal was below background, it was set to 0. If detectable, but below the manufacturer’s lower limit of quantification, it was set to the lower limit of detection.

### 4.7. Statistical analysis

The data were presented as the median (interquartile ranges [IQR]) for continuous variables and as frequency (percentage) for categorical data. Statistical difference between groups was calculated using the Wilcoxon-Mann-Whitney test and Fisher’s exact test to compare continuous variables and dichotomized variables, respectively. Univariable and multivariate logistic regression analysis was performed to assess associations between cfDNA levels and patient characteristics, adjusting for age, gender, self-reported race, and BMI. To evaluate the predictive power of cfDNA and cytokine features for the severity of disease, we constructed a random forest model for SOT patients to classify patients with mild/moderate and severe diseases. The predictive performance of cfDNA profile was evaluated using the receiver operating characteristic (ROC) curve. The ROC curve is plotted as sensitivity (true positive rate) and against 1-specificity (false positive rate) and the accuracy was measured by the area under the curve (AUC). An AUC value of 1 represents a perfect predictive power, whereas an AUC of 0.5 indicates no predictive power. The relative importance for each cfDNA feature was assessed using the mean increase in error rate (decrease in accuracy) over all out-of-bag cross-validated predictions.

To compare the cytokines between patient groups, a linear regression analysis was performed after adjusting for age, gender, and BMI. P-values were obtained from the model and converted to false discovery rates (FDR) using the Benjamini-Hochberg (BH) procedure^78^. To characterize the connection between the cfDNA levels and cytokines, we calculated Spearman’s correlation coefficients among all cfDNA features and cytokines.

To further study the association between cytokines and the origin of tissue damage, we performed a linear regression analysis of the cytokine and cfDNA data. For each cytokine and each cfDNA feature, we fitted a linear regression model using the cytokine as the independent variable and the cfDNA feature as the dependent variable. The t-statistics from the coefficient of linear regression model is used to represent the association between a cytokine and a cfDNA feature. Statistical analyses were carried out in R software version 3.6.3 and GraphPad Prism software version 9. A p-value ≤0.05 and FDR≤0.25 was considered statistically significant;*: FDR ≤ 0.25 and p-value ≤ 0.05, **: FDR ≤ 0.1 and p-value ≤ 0.05, ***: FDR ≤ 0.05 and p-value ≤ 0.05, NS: FDR > 0.25 or p-value > 0.05.

## Supplementary Material

1

## Figures and Tables

**Table 1:** Baseline demographic and clinical characteristics of the SOT patients with COVID-19

Variables		All SOT	Mild-moderate SOT, n=32 (72.7%)	Severe SOT, n=12(27.3%)	P value
Demographic					
Age, years	median (IQR)	54.5 (43.5 – 66.5)	50 (41.5 – 62.5)	62.5 (53.25 – 68.0)	0.030
Sex, n (%)	Male	27 (61.4%)	18 (56.25%)	9 (75%)	
	Female	17 (38.6%)	14 (43.75%)	3 (25%)	0.315
Race/ethinicity, n (%)	Black	16 (36.4%)	11 (34.4%)	5 (41.7%)	
	White	23 (52.3%)	16 (50%)	7 (58.3%)	0.548
	Hispanic	4 (9%)	4 (12.5)	0	
	Others, unknow	1 (2.3%)	1 (3.1%)	0	
BMI (kg/m^2^)	Median (IQR)	28.3 (24.2 – 34.3)	28.15 (23.1 – 33.6)	31.4 (26.7 – 35.3)	0.393
Comorbidities, n (%)	Obesity	10 (22.7%)	6 (18.6%)	4 (33.3%)	0.422
	Diabetes	21 (47.7%)	17 (53.1%)	4 (33.3%)	0.318
	HTN	27 (61.4%)	20 (62.5%)	7 (58.3%)	>0.999
	CHF	7 (15.9%)	6 (18.6%)	1 (8.3%)	0.653
	CAD	9 (20.5%)	6 (18.6%)	3 (25%)	0.687
	Cancer	5 (11.4%)	3 (9.4%)	2 (16.7%)	0.603
	CKD	13 (29.5%)	10 (31.3%)	3 (25%)	>0.999
	Cirrhosis	2 (4.6%)	1 (3.1%)	1 (8.3%)	0.476
	CLD	21 (47.7%)	15 (46.9%)	6 (50%)	>0.999
	HIV	3 (6.8%)	3 (9.4%)	0	0.537
	HCV	5 (11.4%)	5 (15.6%)	0	0.301
	Autoimmunity	3 (6.8%)	3 (9.4%)	0	0.537
Laboratory data	WBC, K/uL	5.29 (3.73 – 6.72)	4.68 (3.63 – 6.11)	7.45 (5.15 – 11.65)	0.015
	ALC, K/uL	0.79 (0.47 – 1.15)	0.85 (0.60 – 1.29)	0.7 (0.38 −1.03)	0.177
	ANC, K/uL	3.82 (2.23 – 4.50)	3.09 (2.05 – 4.23)	5.91 (4.09 – 10.4)	0.002
	Lowest ANC, K/uL	2.40 (1.89 – 3.86)	2.325 (1.86 – 3.74)	2.4 (2.03 – 5.34)	0.443
	Lowest ALC, K/uL	0.52(0.36 – 0.87)	0.49 (0.36 – 0.89)	0.53 (0.25 – 0.6)	0.443
	Creatinine, mg/dL	1.60 (1.23 – 2.10)	1.6 (1.15 – 2.1)	1.9 (1.275 – 2.73)	0.282
	ALP, U/L	76 (58 – 138.5)	75 (56.5 – 139.3)	77 (70 – 140)	0.852
	ALT, U/L	20.5 (12 – 35.5)	21 (12 −33.5)	19 (10–45)	0.982
	AST, U/L	29 (15.5 – 45)	22 (17.75 – 43.5)	36 (15 – 50)	0.363
	D-dimer, mg/L	0.98 (0.36 – 2.49)	0.475 (0.33 – 1.44)	2.57 (1.45 −5.4)	0.001
	CRP, mg/L	2.9 (1.225 – 6.55)	2.8 (1.7 – 6.5)	3.7 (0.9 – 6.7)	0.868
	peak CRP, mg/L	6.9 (2.45 – 11.5)	6.65 (2.275 – 11.1)	8.8 (5.3 – 13.8)	0.292
	max IL-6, pg/mL	31.9 (12.6 – 81.2)	27 (12 – 75.9)	64.6 (26–165)	0.266
	Max TnI, ng/L	0.08 (0.07–0.58)	0.08 (0.07 – 0.94)	0.08 (0.06–0.91)	0.810
SOT types, n(%)	Lung	18 (40.9%)	11 (34.4%)	7 (58.3%)	-
	Kidney	15 (34.1%)	11 (34.4%)	4 (33.3%)	
	Heart	3 (6.8%)	3 (9.4%)	0	
	Liver	7 (15.9%)	6 (18.6%)	1 (8.3%)	
	Liver/kidney	1 (2.3%)	1 (3.1%)	0	
Time posttransplant, years		4 (1–8.5)	4 (1–9)	2.0 (1.25–6.25)	0.479
Hospitalization time, median(IQR)		8 (6–20)	7 (4–10)	26.5 (9.7 – 40)	0.0002
Outcome	Recovered	41	32	9	0.003
	Deceased	3 (25%)	0	3 (25%)	
Pre-admission	Yes	30	21	9	0.727
MMF	No	13	10	3	
MMF held	Yes	27	20	7	0.273
	No	3	1	2	
	Unknown	1	1	0	
COVID-19 treatment	RDV	23 (52.7%)	12	11	0.002
	Convalescent plasma	20 (47.3%)	16	1	0.498
	Crizanlizumab	2	1	1	-
	HCQ	3	3	0	-
	Dex	4	4	0	-
	Toci	1	0	1	-
	None	3	3	0	-

Abbreviations: SOT, solid organ transplant; IQR, interquartile range; BMI, body mass index; HTN, hypertension; CHF, congestive heart failure; CAD, coronary artery disease; CKD, chronic kidney disease; CLD, chronic lung disease; HIV, Human immunodeficiency virus; HCV, Hepatitis C virus; WBC, white blood cell count; ALC, absolute lymphocyte count; ANC, absolute neutrophil count; ALP, alkaline phosphatase; ALT, alanine aminotransferase; AST, aspartate aminotransferase; CRP, C-reactive protein; IL-6, Interleukin-6; TNI troponin I; RDV, remdesivir; HCQ, hydroxychloroquine; DEX, dexamethasone; Toci, tocilizumab.

## Data Availability

The datasets generated in this study study are available from the corresponding author upon reasonable request.
