# Supplementary material for: Integrated cell-free DNA and cytokine analysis uncovers distinct tissue injury and immune response patterns in solid organ transplant recipients with COVID-19
Source: Res Sq. 2022 Jan 20:rs.3.rs-1262270. Preprint. [Version 1] doi: 10.21203/rs.3.rs-1262270/v1 (PMC8786231; doi:10.21203/rs.3.rs-1262270/v1)
Supplement: 1 [file NIHPPRS1262270V1-supplement-1.pdf]

Supplementary Material

Integrated cell-free DNA and cytokine analysis in transplant COVID-19 patients

Andargie et al.

1. Additional Results

1.1. Random forest analysis of cfDNA and cytokine signatures identified SOT patients with severe disease.

To characterize the important cfDNA features that distinguished SOT COVID-19 patients needing ICU care, we performed random forest (RF) analysis (**Suppl.Fig.2a**). The top five cfDNA features at admission that differentiate SOT COVID-19 patients who eventually develop severe disease from those with mild/moderate disease were ncfDNA, neutrophil, monocyte, adipocyte, and erythroblast, respectively. We next performed RF analysis to pinpoint important cytokine features that may affect disease severity (**Suppl.Fig.2b)**. The top five cytokine features that differentiate SOTRs with severe COVID-19 disease were IL-15, IL-16, IL-23p40, TNF-α, and IL-6. The cfDNA RF model showed good performance to discriminate SOT COVID-19 patients into mild/moderate and severe groups, with an area under the curve (AUC) value of 0.815 (95% CI = 0.648 - 0.982). Additionally, the RF model for cytokine features also showed good performance (AUC = 0.796 (95% CI: 0.643-0.948) to distinguish SOT patients who develop severe disease versus mild/moderate. Interestingly, combining the top five cfDNA and cytokine features in the RF analysis improved the performance (AUC = 0.875 (95% CI: 0.765-0.985) to identify SOT patients who progressed to severe COVID-19 (**Suppl.Fig.2c and d)**. These results demonstrate that integrated cfDNA and cytokine analysis improve the performance of RF model to identify SOTRs who subsequently developed severe disease/died.

1.2. cfDNA levels were comparable between lung and other SOT patients.

Given that lung transplant patients have the shortest post-transplant survival of all SOTRs and that respiratory epithelium is the primary site for SARS-CoV-2 infection, we analyzed and compared cfDNA profiles in LTRs with COVID-19 (n=18, 41%) and other SOT types (n=26, 59%). Our data showed no significant difference in total plasma cfDNA levels i.e., mt-cfDNA and ncfDNA, between LTRs with COVID-19 and those with other SOT types ($p>0.05$ and FDR>0.25; **Suppl.Fig.6a and b**). Comparing the cfDNA tissues-of-origin profile, there were no significant differences in the plasma cfDNA levels derived from hematopoietic cells and nonhematopoietic tissue types, including lung, adipocytes, heart and kidney ($p>0.05$ and FDR>0.25; **Suppl.Fig.6c-p**). This adds to the hypothesis that COVID-19 is truly is a systemic disease in SOTRs.

1.3. Correlation of cfDNA profiles and cytokine signatures based on disease severity.

We calculated Spearman's correlation between all biomarkers pairs for patients in different disease severity groups (**Suppl.Fig.4**). We observed three clusters in both mild SOT and Non-SOT COVID-19 patients; however, the size and components of the clusters are different, with the largest cluster that contains 12 cytokines in Non-SOT patients with mild disease. In patients with severe disease, the correlation matrix showed more fragmented correlation patterns, highlighting dysregulated cross-talk between cells and cytokine levels. Thus, the differences in number, size, and components of clusters implied distinct tissue injury and cytokine patterns between SOTRs with COVID-19 and Non-SOT COVID-19 patients, as well as disease severity. Likewise, a two-way hierarchical cluster heatmap generated showed divergent tissue injury patterns and cytokine response

correlated with disease severity in both SOT and Non-SOT COVID-19 patients (**Suppl.Fig.5**). The Spearman's correlation also revealed that cfDNA profiles were correlated with altered cytokine levels both in mild/moderate and severe SOT patients, including IL-8, IL-18, TNF-α, IL-12p70, and IL-2Ra. In mild/moderate SOT patients, monocyte-derived cfDNA correlated with multiple cytokines (IL-6, IL-8, IL-18, IL-10, IP-10, IL-4, IL-15, IL-17A, GM-CSF, and MCP-2). Whereas in severe SOT patients adipocyte-derived cfDNA showed the most pronounced association with cytokines (IL-5, TNF-α, Eotaxin, MIP-1α, MIP-1β, IL-23p40, and IL-17A) followed by lung (MCP-1, IL-2Ra, and IL-18) and monocytes (MDC, IL-12p70, and IL-23p40). Interestingly, the proinflammatory cytokine/chemokine IL-18 and IL-8 were positively correlated with mtcfDNA (a DAMP) in SOT COVID-19 patients with severe disease. Moreover, SOT patients with severe disease displayed a strong correlation between cytokine levels and non-hematopoietic tissue-derived cfDNA. Conversely, in Non-SOT patients with COVID-19, fewer interactions were observed between cytokine levels and cfDNA profile, including in patients with severe COVID-19 disease. Overall, these observations provide evidence of excessive cfDNA and exacerbated cytokine response interaction in SOTRs with COVID-19.

1.4. Association between cfDNA profiles and known clinical and biochemical markers.

We further investigated the association of cfDNA levels with clinical parameters and visualized it as a heatmap (Supp.Fig.8). Unsupervised correlation clustering analysis revealed that D-dimer was co-clustered with total (ncfDNA) and tissue-specific cfDNA levels (neutrophils, adipocytes, monocytes, ddcfDNA, NK-cells, and erythroblasts) and liver function test enzymes (ALT and AST) with hepatocyte-derived cfDNA. We then

assess how the tissue injury pattern relates to the age of the patients, a key determinant of poor COVID-19 outcomes. Our analysis demonstrated that age was positively correlated with total nuclear-derived cfDNA (r=0.38, p=0.010) and tissue-specific cfDNA derived from monocytes (r=0.34, p=0.026), erythroblasts (r=0.35, p=0.019), adipocytes (r=0.30, p=0.046), pancreas (r=0.34, p=0.025), ddcfDNA (r=0.33, p=0.043)and marginally correlated with cfDNA derived from neutrophil (r=0.27, p=0.077). These results indicate that cfDNA levels were influenced by age and the increased cfDNA levels were more pronounced in older SOT COVID-19 patients. Total nuclear-origin and tissue-specific cfDNA were not associated with sex in our cohort. Hospitalization length of stay was also significantly associated with global nuclear (r=0.44, p=0.003) and tissue-specific cfDNA levels derived from monocyte (r=0.38, p=0.012), neutrophil (r=0.45, p=0.002), pancreas (r=0.37, p=0.019) and ddcfDNA (r=0.42, p=0.011).

Correlation analysis also revealed that cfDNA levels were correlated with clinically established markers (p<0.05 and FDR<0.25), as shown between neutrophil-derived cfDNA and ANC (r=0.45), NK cells-derived cfDNA and ALC (r=0.38), and hepatocyte-derived cfDNA and liver function tests i.e., AST (r=0.70) and ALT (r=0.64). The serum D-dimer level, an indirect marker of fibrinolysis and fibrin turnover, was strongly correlated with circulating ncfDNA (r=0.69) and cfDNA derived from monocyte (r=0.51), neutrophil (r=0.63), erythroblast (r=0.53), adipocyte (r=0.54), pancreas (r=0.33) and ddcfDNA (r=0.38). We also observed peak levels of CRP, a marker of inflammation, were significantly correlated with admission circulating level of ncfDNA (r=0.33), hepatocyte-

derived cfDNA (r=0.41) and ddcfDNA (r=0.38). Likewise, peak levels of IL-6 were significantly associated ncfDNA (r=0.39) and tissue-specific cfDNA released from NK cell (r=0.36), hepatocyte (r=0.50), pancreas (r=0.53), squamous epithelium (r=0.32), and kidney (r=0.55). Overall, these results indicate the potential utility of cfDNA as a comprehensive noninvasive "liquid biopsy" in transplant patients with COVID-19.

1.5. Longitudinal cfDNA and cytokine profiles in SOTRs with COVID-19.

To better understand the kinetics cfDNA profiles and cytokine levels, a subset of 5 SOT COVID-19 (4 with mild/moderate infection and 1 with severe infection who died) and 4 Non-SOT COVID-19 patients underwent longitudinal blood sample collection (**Fig.11**). We analyzed absolute tissue-specific cfDNA composition and cytokine profile kinetics. In a LTR COVID-19 patient who eventually died, baseline plasma ncfDNA, ddcfdNA, mtcfDNA cfDNA level were 39, 216 cp/mL, 1.44%, and 78,5248 cp/mL, respectively. On day 3 after admission, the plasma ncfDNA and mtcfDNA increased approximately 5 and 20 fold compared to day 1, respectively. The ncfDNA continued to increase from day 3 to day 7, and the levels of the total cfDNA remained elevated above the median cfDNA levels for severe patients until the patient died. In contrast, the total and tissue-specific cfDNA levels gradually decreased with time in SOT patients with mild/moderate disease who eventually recovered. Similarly, proinflammatory cytokines were markedly increased over time in patients who eventually died. These findings suggest that SOT recipients, like Non-SOT patients (**Suppl.Fig.7**), release excessive cfDNA amounts and display an exuberant inflammatory response following SARS-CoV2 infection[27,31,32].

1 ## 2. Supplementary Table

2 Suppl Table 1: Comparison of SOT and Non-SOT COVID-19 patients.

Variables		Non-SOT, n=40	SOT, n=44	P value
Demographic				
Age, years	Median (IQR)	59.5 (39.25 – 70.5)	54.5 (43.5 - 66.5)	0.651
Sex, n (%)	Male	20 (50%)	27 (61.4%)	0.380
	Female	20 (50%)	17 (38.6%)	
Race/ethinicity, n (%)	Black/African American	18 (45%)	16 (36.4%)	0.001
	White	6 (15%)	23 (52.3%)	
	Hispanic	11 (27.5%)	4 (9%)	
	Others, unknown	5 (12.5%)	1 (2.3%)	
BMI (kg/m^2)	Median (IQR)	30.3 (24.1 – 38.2)	28.3 (24.2 - 34.3)	0.376
Comorbidities, n (%)	Obesity	12 (30%)	10 (22.7%)	0.469
	Diabetes	17 (42.5%)	21 (47.7%)	0.666
	HTN	21 (52.5%)	27 (61.4%)	0.509
	CHF	7 (17.5%)	7 (15.9%)	>0.999
	CAD	1 (2.5%)	9 (20.5%)	0.016
	HIV	2 (5%)	3 (6.8%)	>0.999

	HCV	1 (2.5%)	5 (11.4%)	0.205
	CLD	9 (22.5%)	21 (47.7%)	0.023
	Cancer	6 (15%)	5 (11.4%)	0.750
	Autoimmunity	3 (7.5%)	3 (6.8%)	>0.999
Laboratory data	WBC, K/uL	6.85 (4.98 -8.60)	5.29 (3.73-6.72)	0.020
	ALC, K/uL	1.18 (0.74 - 1.77)	0.79 (0.47 - 1.15)	0.010
	ANC, K/uL	4.42 (3.12 – 7.41)	3.82 (2.23 - 5.00)	0.033
	D-dimer, mg/L	1.35 (0.74 -1.73)	0.98 (0.36 - 2.49)	0.112
	CRP, mg/L	5.5 (3 -11.15)	2.9 (1.225 - 6.55)	0.013
Hospitalization time, median(IQR)		10 (4 – 23)	8 (6-20)	0.965
Max WHO severity scale, median (IQR)		4 (3-5)	4 (3-7)	0.087
Outcome	Recovered	36	41	0.704
	Deceased	4	3 (25%)	

3. Supplementary Figures

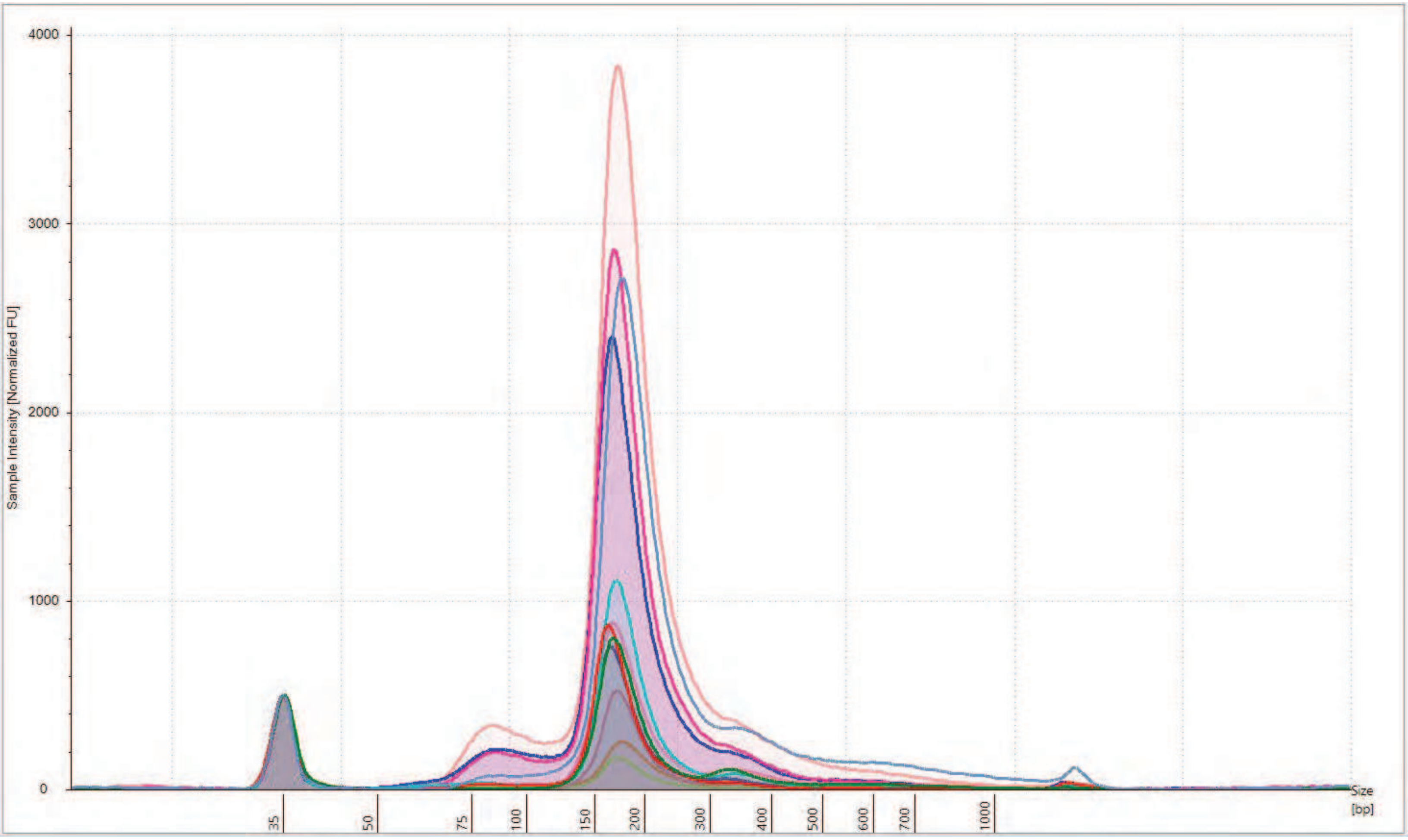

4000
3000
2000
1000
0
Sample Intensity [Normalized FU]
Size
[bp]
35
50
75
100
150
200
300
400
500
600
700
1000

Supplementary Fig. 1. Fragment size distribution of cfDNA in representative samples. A prominent peak length around ~167 bp indicates a good quality of cfDNA.

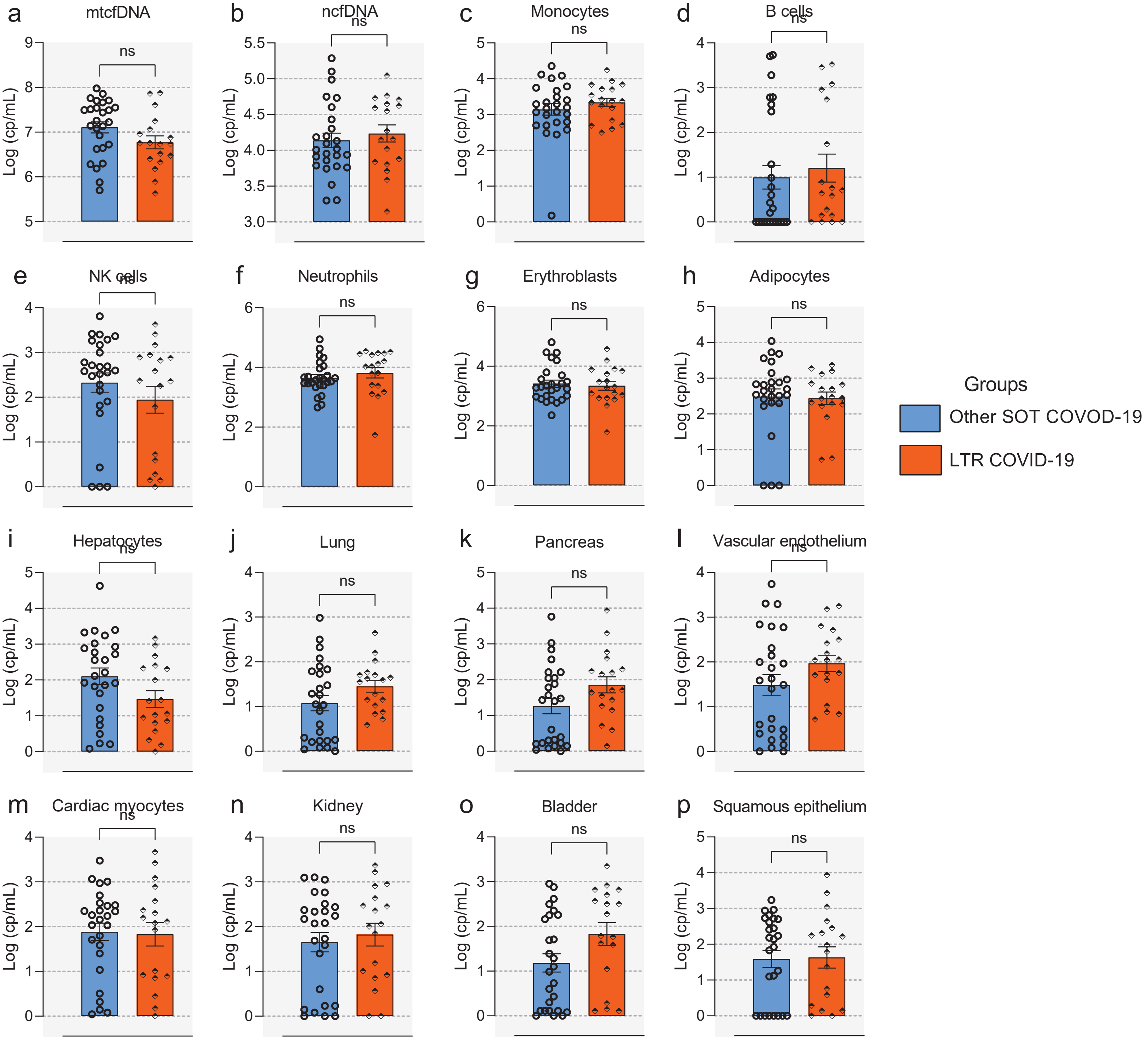

a
mtcfDNA
b
ncfDNA
c
Monocytes
d
B cells
e
NK cells
f
Neutrophils
g
Erythroblasts
h
Adipocytes
i
Hepatocytes
j
Lung
k
Pancreas
l
Vascular endothelium
m
Cardiac myocytes
n
Kidney
o
Bladder
p
Squamous epithelium
ns
Log (cp/mL)
Groups
Other SOT COVOD-19
LTR COVID-19

Supplementary Fig. 2. Similar cfDNA tissue injury pattern among SOT types. Comparisons of absolute total mtcfDNA (**a**) ncfDNA and mtcfDNA (**b**) and tissue-specific cfDNA levels derived monocytes (**c**), B cells (**d**), NK cells (**e**), neutrophils (**f**), erythroblasts (**g**), vascular endothelium (**h**), adipocytes (**i**), hepatocytes (**j**), lung (**k**), pancreas (**l**), cardiac myocytes (**m**), kidney (**n**), bladder (**o**), and squamous epithelium (**p**) between lung transplant recipients with COVID-19 (n=18) other SOT types (n=26). Statistical significance was determined by the Mann–Whitney test. Adjusted p values are reported (for multiple comparison and demographic factors (age, sex, and BMI). A p-value ≤0.05 and FDR≤0.25 was considered statistically significant;*: FDR ≤ 0.25 and p-value ≤ 0.05, **: FDR ≤ 0.1 and p-value ≤ 0.05, ***: FDR ≤ 0.05 and p-value ≤ 0.05, NS: FDR > 0.25 or p-value > 0.05.

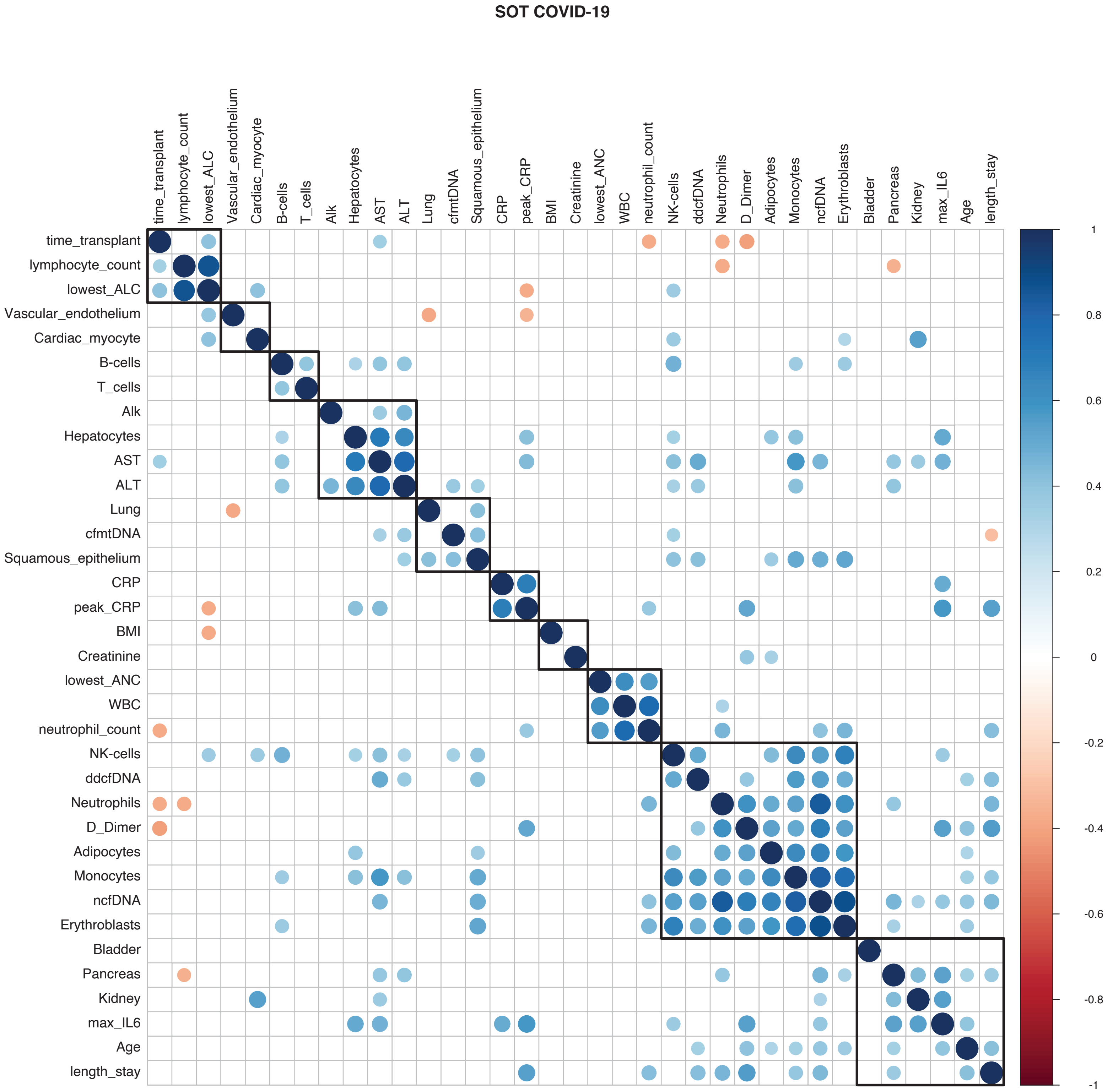
SOT COVID-19
time_transplant
lymphocyte_count
lowest_ALC
Vascular_endothelium
Cardiac_myocyte
B-cells
T_cells
Alk
Hepatocytes
AST
ALT
Lung
cfmtDNA
Squamous_epithelium
CRP
peak_CRP
BMI
Creatinine
lowest_ANC
WBC
neutrophil_count
NK-cells
ddcfDNA
Neutrophils
D_Dimer
Adipocytes
Monocytes
ncfDNA
Erythroblasts
Bladder
Pancreas
Kidney
max_IL6
Age
length_stay
1
0.8
0.6
0.4
0.2
0
-0.2
-0.4
-0.6
-0.8
-1

Supplementary Fig. 3. Circulating cfDNA levels associated with conventional clinical and biochemical profiles. Heat map between cfDNA and continuous conventional clinical and biochemical profiles conducted upon a Spearman correlation in SOTRs with COVID-19. A p-value ≤0.05 and FDR≤0.25 was considered significant;*: FDR ≤ 0.25 and p-value ≤ 0.05, **: FDR ≤ 0.1 and p-value ≤ 0.05, ***: FDR ≤ 0.05 and p-value ≤ 0.05, NS: FDR > 0.25 or p-value > 0.05.

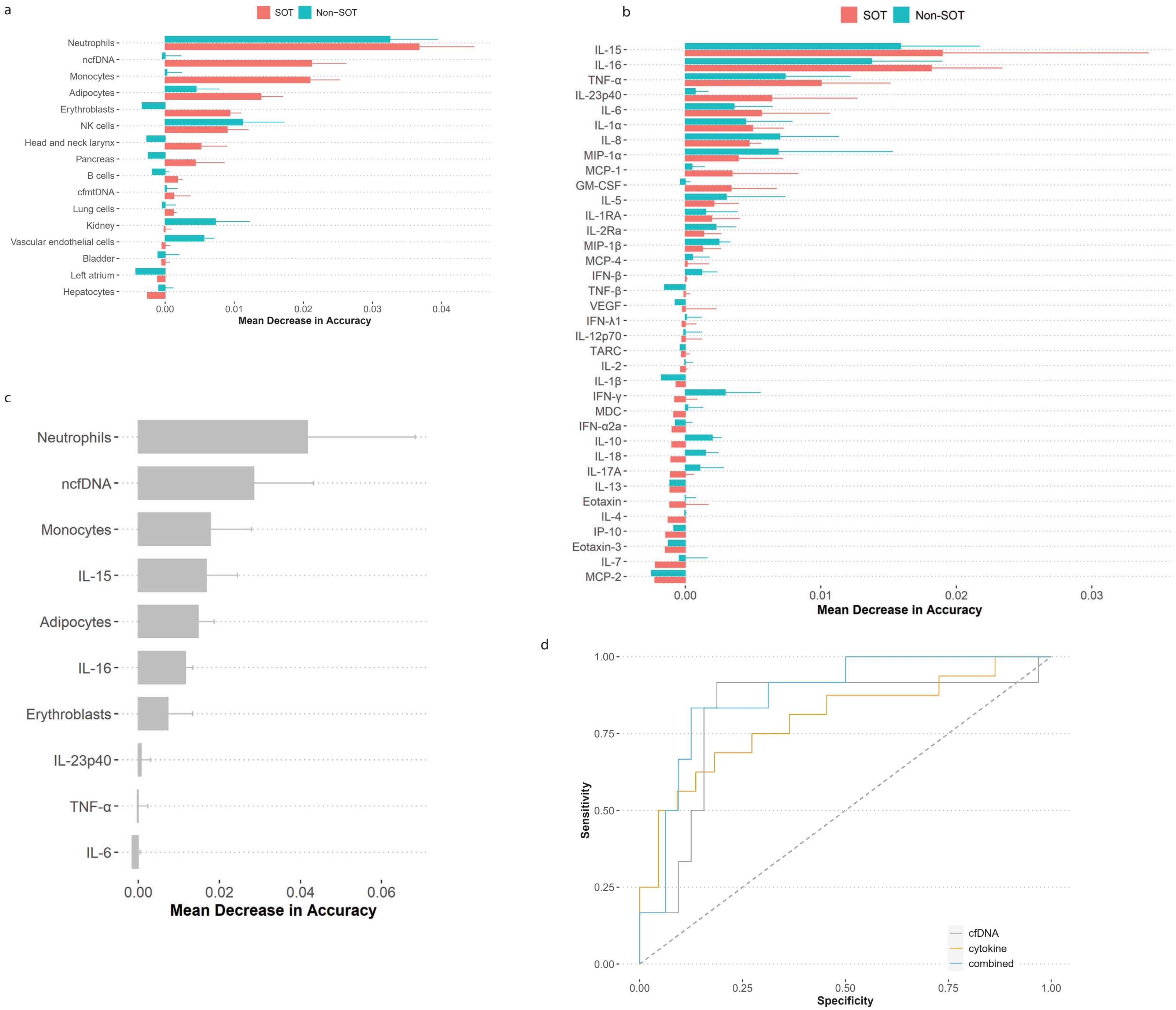
a
SOT
Non−SOT
Neutrophils
ncfDNA
Monocytes
Adipocytes
Erythroblasts
NK cells
Head and neck larynx
Pancreas
B cells
cfmtDNA
Lung cells
Kidney
Vascular endothelial cells
Bladder
Left atrium
Hepatocytes
0.00
0.01
0.02
0.03
0.04
Mean Decrease in Accuracy
b
SOT
Non−SOT
IL-15
IL-16
TNF-α
IL-23p40
IL-6
IL-1α
IL-8
MIP-1α
MCP-1
GM-CSF
IL-5
IL-1RA
IL-2Ra
MIP-1β
MCP-4
IFN-β
TNF-β
VEGF
IFN-λ1
IL-12p70
TARC
IL-2
IL-1β
IFN-γ
MDC
IFN-α2a
IL-10
IL-18
IL-17A
IL-13
Eotaxin
IL-4
IP-10
Eotaxin-3
IL-7
MCP-2
0.00
0.01
0.02
0.03
Mean Decrease in Accuracy
c
Neutrophils
ncfDNA
Monocytes
IL-15
Adipocytes
IL-16
Erythroblasts
IL-23p40
TNF-α
IL-6
0.00
0.02
0.04
0.06
Mean Decrease in Accuracy
d
1.00
0.75
0.50
0.25
0.00
Sensitivity
0.00
0.25
0.50
0.75
1.00
Specificity
cfDNA
cytokine
combined

Supplementary Fig. 4. Plasma cfDNA and cytokine signatures identify patients with severe disease. (a) Rank of important cfDNA features to identify patients with severe disease in SOT and Non-SOT COVID-19 patients based on random forest model (b) Rank of important cytokines features to identify patients with severe disease in SOT and Non-SOT COVID-19 patients based on random forest model. (c) Top five cfDNA and cytokine features to identify SOT patients with severe disease. (d) Performance of top five cfDNA and cytokine features to identify severe SOT patients from mild/moderate groups.

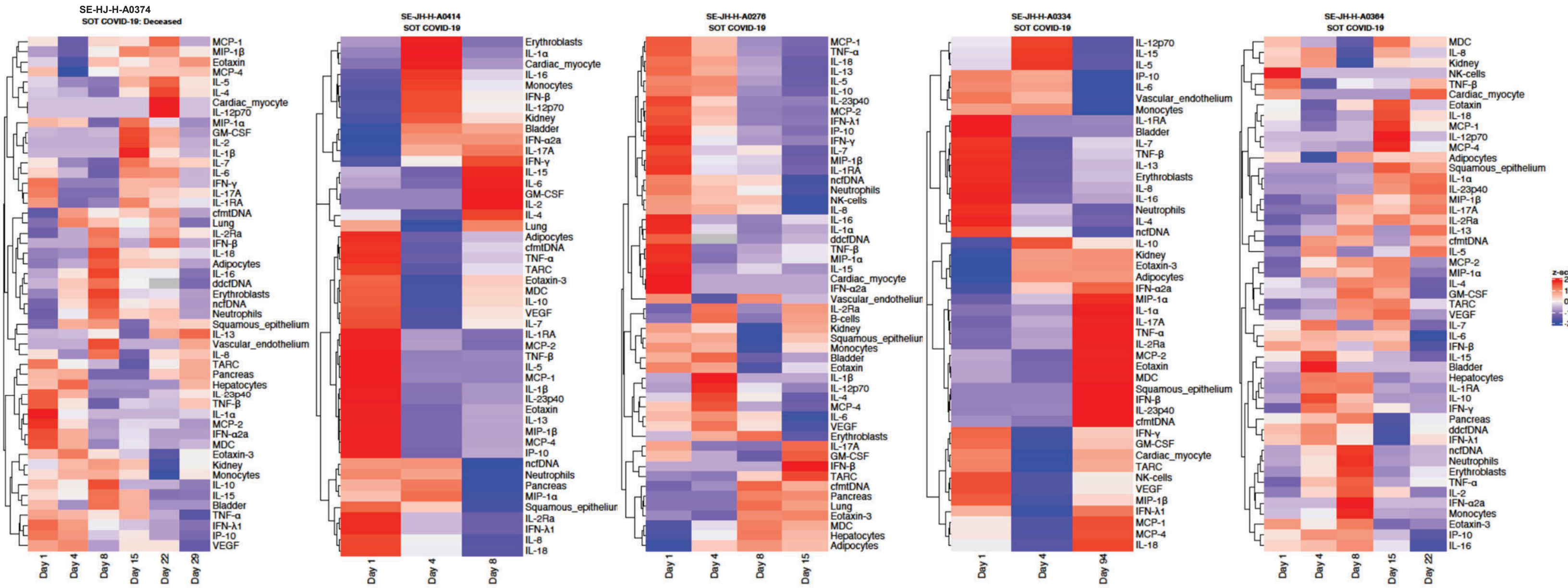
SE-HJ-H-A0374
SOT COVID-19: Deceased
Day 1
Day 4
Day 8
Day 15
Day 22
Day 29
SE-JH-H-A0414
SOT COVID-19
Day 1
Day 4
Day 8
SE-JH-H-A0276
SOT COVID-19
Day 1
Day 4
Day 8
Day 15
SE-JH-H-A0334
SOT COVID-19
Day 1
Day 4
Day 94
SE-JH-H-A0364
SOT COVID-19
Day 1
Day 4
Day 8
Day 15
Day 22

Supplementary Fig. 5. Heatmap of longitudinal cfDNA level and cytokine response of prototype SOT COVID-19 patients. Changes in cfDNA measures and cytokine levels over time for SOT patients with severe disease who died (Deceased); with unresolved tissue injury and cytokine pattern (a) and a gradually decreasing cfDNA and cytokine levels in SOTRs who recovered (b-e).

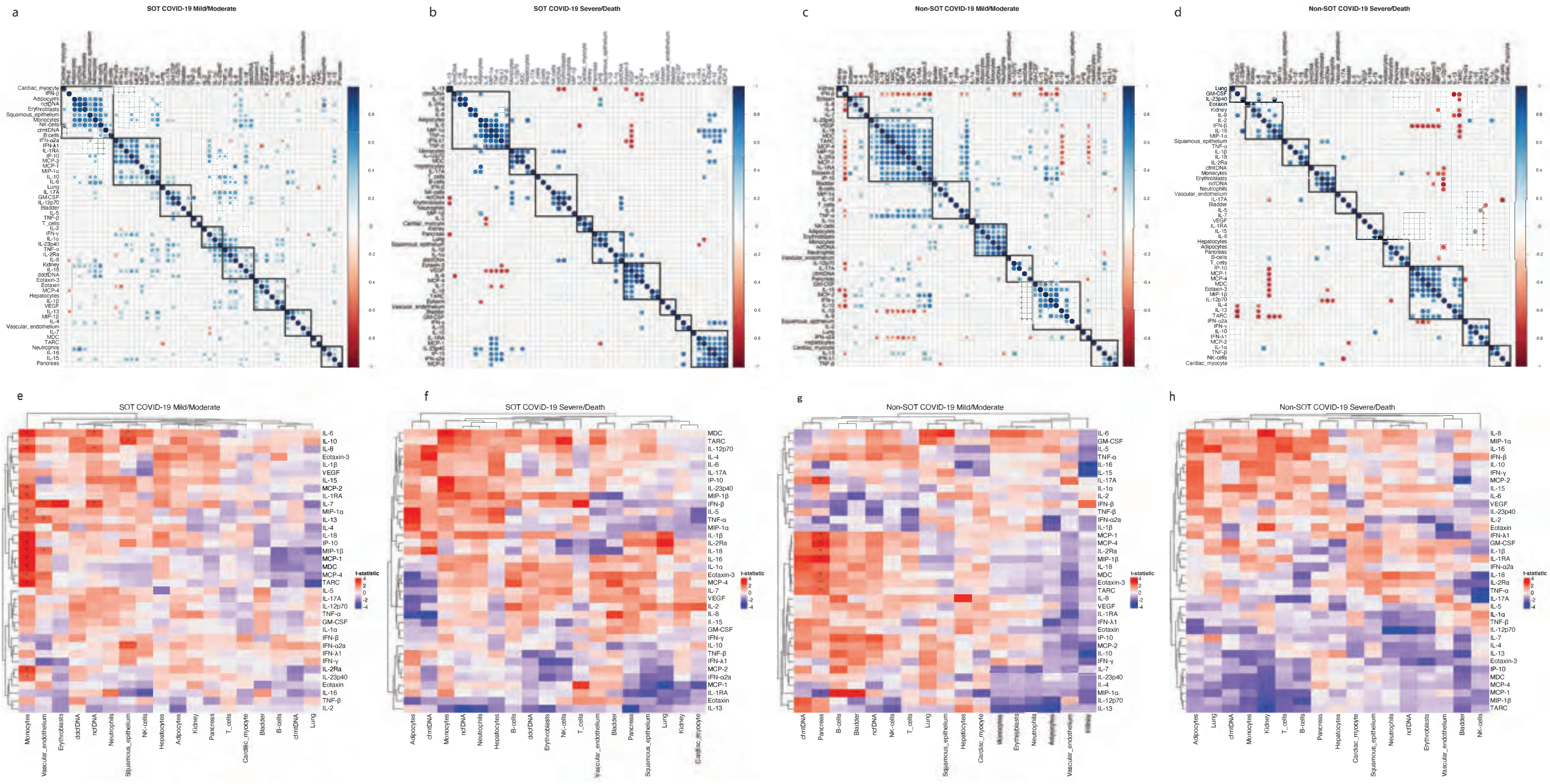
a
SOT COVID-19 Mild/Moderate
b
SOT COVID-19 Severe/Death
c
Non-SOT COVID-19 Mild/Moderate
d
Non-SOT COVID-19 Severe/Death
e
SOT COVID-19 Mild/Moderate
f
SOT COVID-19 Severe/Death
g
Non-SOT COVID-19 Mild/Moderate
h
Non-SOT COVID-19 Severe/Death
t-statistic

Supplementary Fig. 6. Association between cfDNA and cytokine profiles in SOT COVID-19 patients subgroups based on disease severity. Pearson correlation matrix between cfDNA and cytokines/chemokines profiles in SOTRs with mild/moderate disease (n=32) (a) and severe disease (n=12) (b). Hierarchical Clustering Heatmap of circulating cfDNA and cytokine profiles in SOT patients with Mild/moderate (c) and severe COVID-19 disease (d). Spearman correlations test and linear regression analysis were performed to determine statistical significance. A p-value ≤0.05 and FDR≤0.25 was considered significant;*: FDR ≤ 0.25 and p-value ≤ 0.05, **: FDR ≤ 0.1 and p-value ≤ 0.05, ***: FDR ≤ 0.05 and p-value ≤ 0.05, NS: FDR > 0.25 or p-value > 0.05. Adjusted p values are reported (for multiple comparison and demographic factors (age, sex, and BMI).

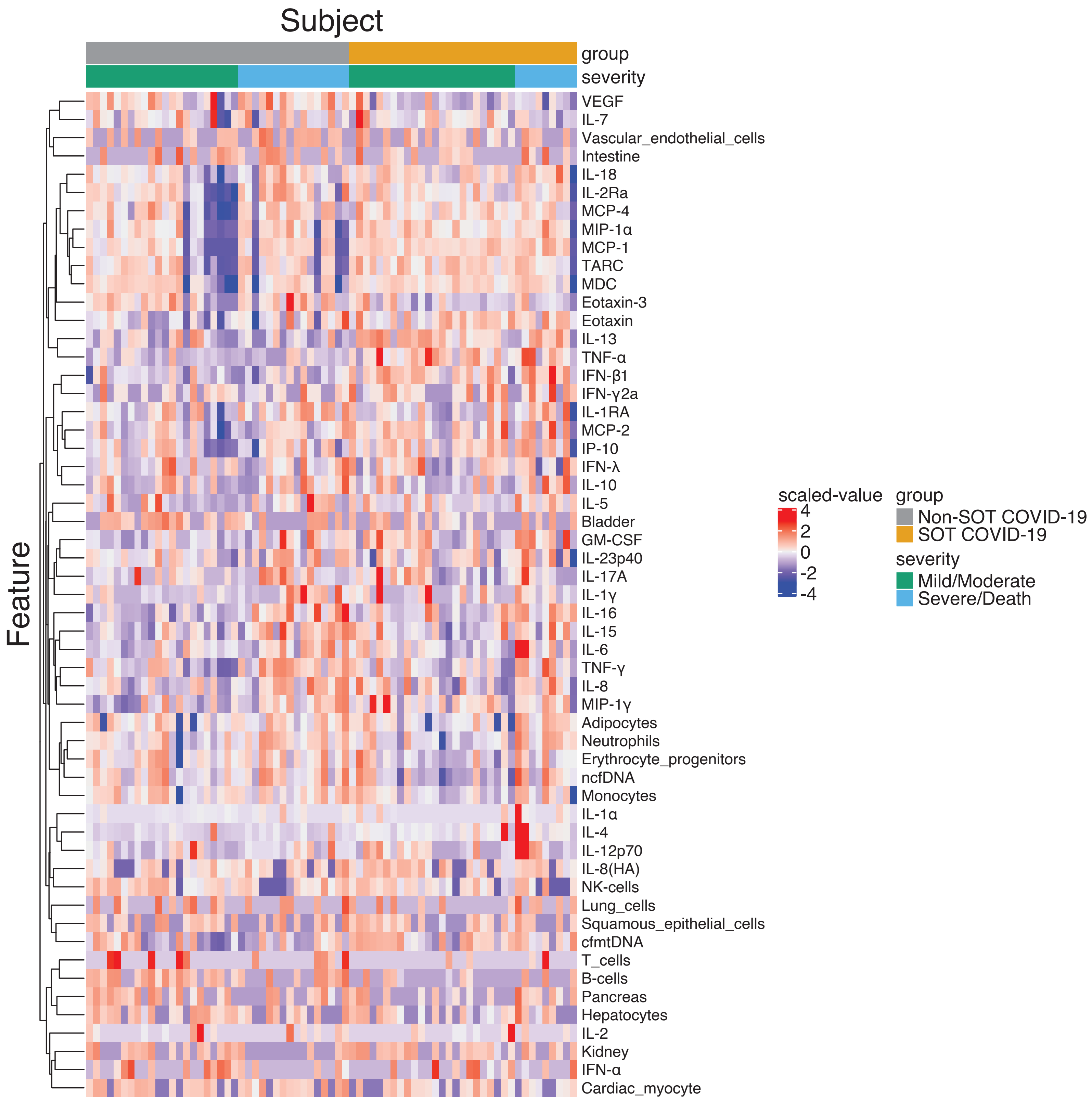

Subject
group
severity
Feature
VEGF
IL-7
Vascular_endothelial_cells
Intestine
IL-18
IL-2Ra
MCP-4
MIP-1α
MCP-1
TARC
MDC
Eotaxin-3
Eotaxin
IL-13
TNF-α
IFN-β1
IFN-γ2a
IL-1RA
MCP-2
IP-10
IFN-λ
IL-10
IL-5
Bladder
GM-CSF
IL-23p40
IL-17A
IL-1γ
IL-16
IL-15
IL-6
TNF-γ
IL-8
MIP-1γ
Adipocytes
Neutrophils
Erythrocyte_progenitors
ncfDNA
Monocytes
IL-1α
IL-4
IL-12p70
IL-8(HA)
NK-cells
Lung_cells
Squamous_epithelial_cells
cfmtDNA
T_cells
B-cells
Pancreas
Hepatocytes
IL-2
Kidney
IFN-α
Cardiac_myocyte
scaled-value
4
2
0
-2
-4
group
Non-SOT COVID-19
SOT COVID-19
severity
Mild/Moderate
Severe/Death

Supplementary Fig. 7. Heatmap showing divergent tissue injury patterns and cytokine response that correlated with disease severity in SOT COVID-19 patients**.**

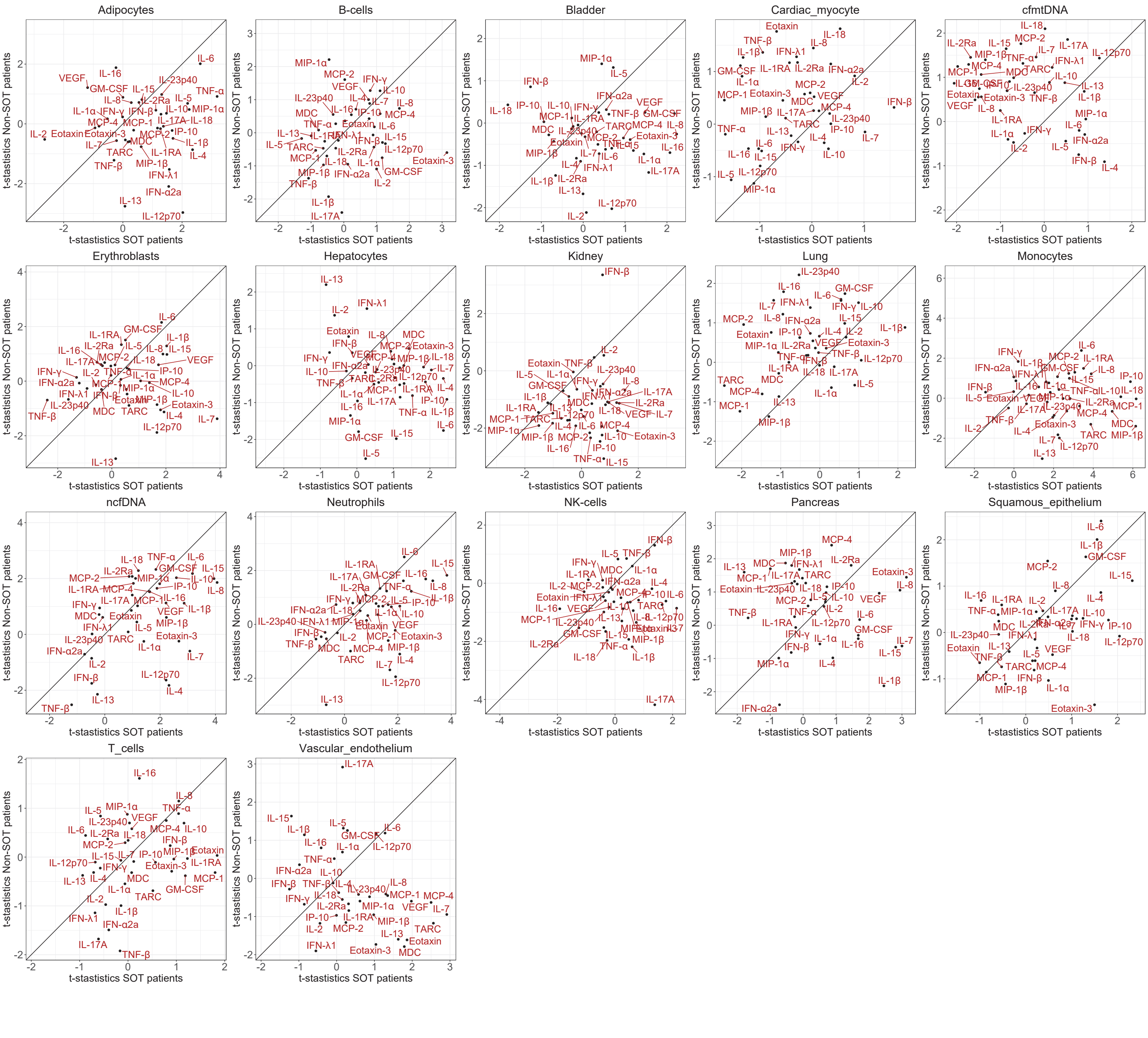

Adipocytes
B-cells
Bladder
Cardiac_myocyte
cfmtDNA
Erythroblasts
Hepatocytes
Kidney
Lung
Monocytes
ncfDNA
Neutrophils
NK-cells
Pancreas
Squamous_epithelium
T_cells
Vascular_endothelium
t-stastistics SOT patients
t-statistics Non-SOT patients

Supplementary Fig. 8. t-statistics scatterplot plots between cfDNA and cytokine levels in SOT patients with COVID-19. Linear regression analysis between cfDNA features and cytokine profile was conducted.

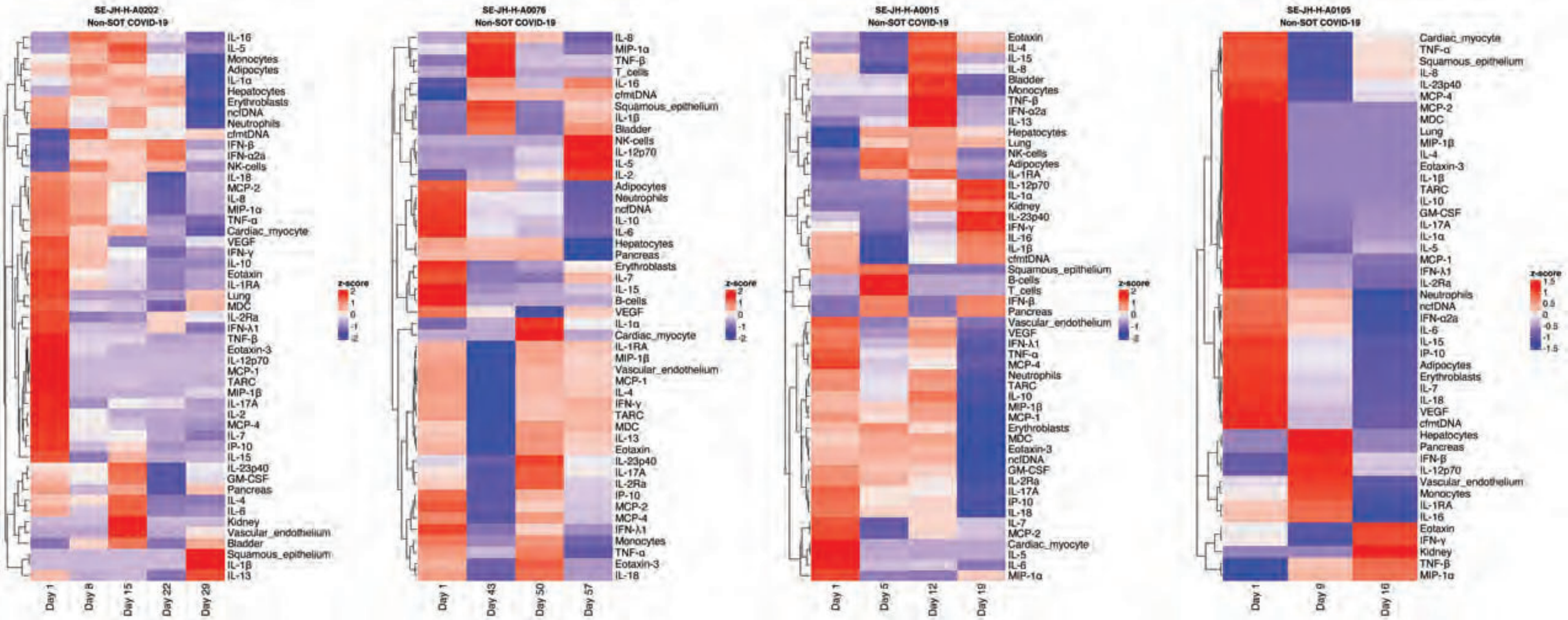
SE-JH-H-A0202
Non-SOT COVID-19
IL-16
IL-5
Monocytes
Adipocytes
IL-1α
Hepatocytes
Erythroblasts
ncfDNA
Neutrophils
cfmtDNA
IFN-β
IFN-α2a
NK-cells
IL-18
MCP-2
IL-8
MIP-1α
TNF-α
Cardiac_myocyte
VEGF
IFN-γ
IL-10
Eotaxin
IL-1RA
Lung
MDC
IL-2Ra
IFN-λ1
TNF-β
Eotaxin-3
IL-12p70
MCP-1
TARC
MIP-1β
IL-17A
IL-2
MCP-4
IL-7
IP-10
IL-15
IL-23p40
GM-CSF
Pancreas
IL-4
IL-6
Kidney
Vascular_endothelium
Bladder
Squamous_epithelium
IL-1β
IL-13
Day 1
Day 8
Day 15
Day 22
Day 29
z-score
2
1
0
-1
-2
SE-JH-H-A0076
Non-SOT COVID-19
IL-8
MIP-1α
TNF-β
T_cells
IL-16
cfmtDNA
Squamous_epithelium
IL-1β
Bladder
NK-cells
IL-12p70
IL-5
IL-2
Adipocytes
Neutrophils
ncfDNA
IL-10
IL-6
Hepatocytes
Pancreas
Erythroblasts
IL-7
IL-15
B-cells
VEGF
IL-1α
Cardiac_myocyte
IL-1RA
MIP-1β
Vascular_endothelium
MCP-1
IL-4
IFN-γ
TARC
MDC
IL-13
Eotaxin
IL-23p40
IL-17A
IL-2Ra
IP-10
MCP-2
MCP-4
IFN-λ1
Monocytes
TNF-α
Eotaxin-3
IL-18
Day 1
Day 43
Day 50
Day 57
z-score
2
1
0
-1
-2
SE-JH-H-A0015
Non-SOT COVID-19
Eotaxin
IL-4
IL-15
IL-8
Bladder
Monocytes
TNF-β
IFN-α2a
IL-13
Hepatocytes
Lung
NK-cells
Adipocytes
IL-1RA
IL-12p70
IL-1α
Kidney
IL-23p40
IFN-γ
IL-16
IL-1β
cfmtDNA
Squamous_epithelium
B-cells
T_cells
IFN-β
Pancreas
Vascular_endothelium
VEGF
IFN-λ1
TNF-α
MCP-4
Neutrophils
TARC
IL-10
MIP-1β
MCP-1
Erythroblasts
MDC
Eotaxin-3
ncfDNA
GM-CSF
IL-2Ra
IL-17A
IP-10
IL-18
IL-7
MCP-2
Cardiac_myocyte
IL-5
IL-6
MIP-1α
Day 1
Day 5
Day 12
Day 19
z-score
2
1
0
-1
-2
SE-JH-H-A0105
Non-SOT COVID-19
Cardiac_myocyte
TNF-α
Squamous_epithelium
IL-8
IL-23p40
MCP-4
MCP-2
MDC
Lung
MIP-1β
IL-4
Eotaxin-3
IL-1β
TARC
IL-10
GM-CSF
IL-17A
IL-1α
IL-5
MCP-1
IFN-λ1
IL-2Ra
Neutrophils
ncfDNA
IFN-α2a
IL-6
IL-15
IP-10
Adipocytes
Erythroblasts
IL-7
IL-18
VEGF
cfmtDNA
Hepatocytes
Pancreas
IFN-β
IL-12p70
Vascular_endothelium
Monocytes
IL-1RA
IL-16
Eotaxin
IFN-γ
Kidney
TNF-β
MIP-1α
Day 1
Day 9
Day 16
z-score
1.5
1
0.5
0
-0.5
-1
-1.5

Supplementary Fig. 9. Heatmap of longitudinal cfDNA level and cytokine response of prototype Non-SOT COVID-19 patients.
